# Structural Basis of Response Regulator Inhibition by a Bacterial Anti-Activator Protein

**DOI:** 10.1371/journal.pbio.1001226

**Published:** 2011-12-27

**Authors:** Melinda D. Baker, Matthew B. Neiditch

**Affiliations:** Department of Microbiology and Molecular Genetics, UMDNJ–New Jersey Medical School, Newark, New Jersey, United States of America; UMDNJ/Robert Wood Johnson Medical School/HHMI, United States of America

## Abstract

The complex interplay between the response regulator ComA, the anti-activator RapF, and the signaling peptide PhrF controls competence development in *Bacillus subtilis*. More specifically, ComA drives the expression of genetic competence genes, while RapF inhibits the interaction of ComA with its target promoters. The signaling peptide PhrF accumulates at high cell density and upregulates genetic competence by antagonizing the interaction of RapF and ComA. How RapF functions mechanistically to inhibit ComA activity and how PhrF in turn antagonizes the RapF-ComA interaction were unknown. Here we present the X-ray crystal structure of RapF in complex with the ComA DNA binding domain. Along with biochemical and genetic studies, the X-ray crystal structure reveals how RapF mechanistically regulates ComA function. Interestingly, we found that a RapF surface mimics DNA to block ComA binding to its target promoters. Furthermore, RapF is a monomer either alone or in complex with PhrF, and it undergoes a conformational change upon binding to PhrF, which likely causes the dissociation of ComA from the RapF-ComA complex. Finally, we compare the structure of RapF complexed with the ComA DNA binding domain and the structure of RapH complexed with Spo0F. This comparison reveals that RapF and RapH have strikingly similar overall structures, and that they have evolved different, non-overlapping surfaces to interact with diverse cellular targets. To our knowledge, the data presented here reveal the first atomic level insight into the inhibition of response regulator DNA binding by an anti-activator. Compounds that affect the interaction of Rap and Rap-like proteins with their target domains could serve to regulate medically and commercially important phenotypes in numerous *Bacillus* species, such as sporulation in *B. anthracis* and sporulation and the production of Cry protein endotoxin in *B. thuringiensis*.

## Abstract 2

Structure-function studies reveal that Rap proteins have distinct, nonoverlapping surfaces that interact with different cellular targets, and that for antiactivator RapF, one surface mimics DNA to bind a response regulator DNA binding domain, thereby sterically preventing the activity of this transcription transactivator.

## Author Summary

Upon phosphorylation, bacterial proteins called response regulators bind to DNA promoters and activate or repress transcription. These response regulators are themselves regulated by anti-activator proteins, which can control response regulator activity without altering their phosphorylation state. We have determined the X-ray crystal structure of the anti-activator RapF complexed with the DNA-binding domain of the response regulator ComA. Our structure-function studies show that RapF disrupts the binding of ComA to DNA using a two-pronged mechanism. First, a RapF surface mimics DNA, and this DNA-like surface binds to nearly all of the ComA DNA-binding residues, thus blocking ComA's interaction with DNA. Second, RapF inhibits ComA dimerization. RapF is also regulated by the PhrF peptide; we find that the RapF-ComA interaction surface is distant from the proposed PhrF binding site. Furthermore, we found that RapF undergoes a conformational change upon binding to PhrF, which likely causes its dissociation from ComA. From these observations, we conclude that PhrF binding to RapF allosterically triggers its dissociation from ComA. Finally, we compared the RapF/ComA DNA-binding domain complex structure with the structure of another response regulator, Spo0F, complexed with the phosphatase RapH. This reveals that while RapF and RapH are structurally similar, they have evolved distinct, non-overlapping surfaces to interact with their different cellular targets.

## Introduction

Two-component signaling systems, consisting of a sensor histidine kinase and a response regulator transcription factor, are the principal mechanism of signal transduction in bacteria [1]. Upon phosphorylation of their receiver (REC) domains, response regulators bind target DNA promoters and activate or repress transcription. A number of auxiliary factors have garnered significant attention because they function as anti-activators that inhibit response regulator function without affecting their phosphorylation state. These proteins inhibit the interaction of response regulators with their target promoters [2]–[5] or RNA polymerase [6],[7]. To our knowledge, the X-ray crystallographic, biochemical, and genetic results presented here show for the first time how an anti-activator structurally inhibits the binding of a response regulator to DNA.

The Rap proteins are a family of auxiliary factors that have been most thoroughly studied in *Bacillus subtilis*, where they regulate two-component and phosphorelay signal transduction. Despite their relatively high sequence similarity, different Rap proteins exert their influence on these pathways via disparate activities [5]. Rap proteins ultimately influence diverse *B. subtilis* developmental and cellular differentiation processes including sporulation, genetic competence, and biofilm formation, as well as the production of secreted enzymes and the movement of a conjugative transposon [8],[9].

**Figure 1:**
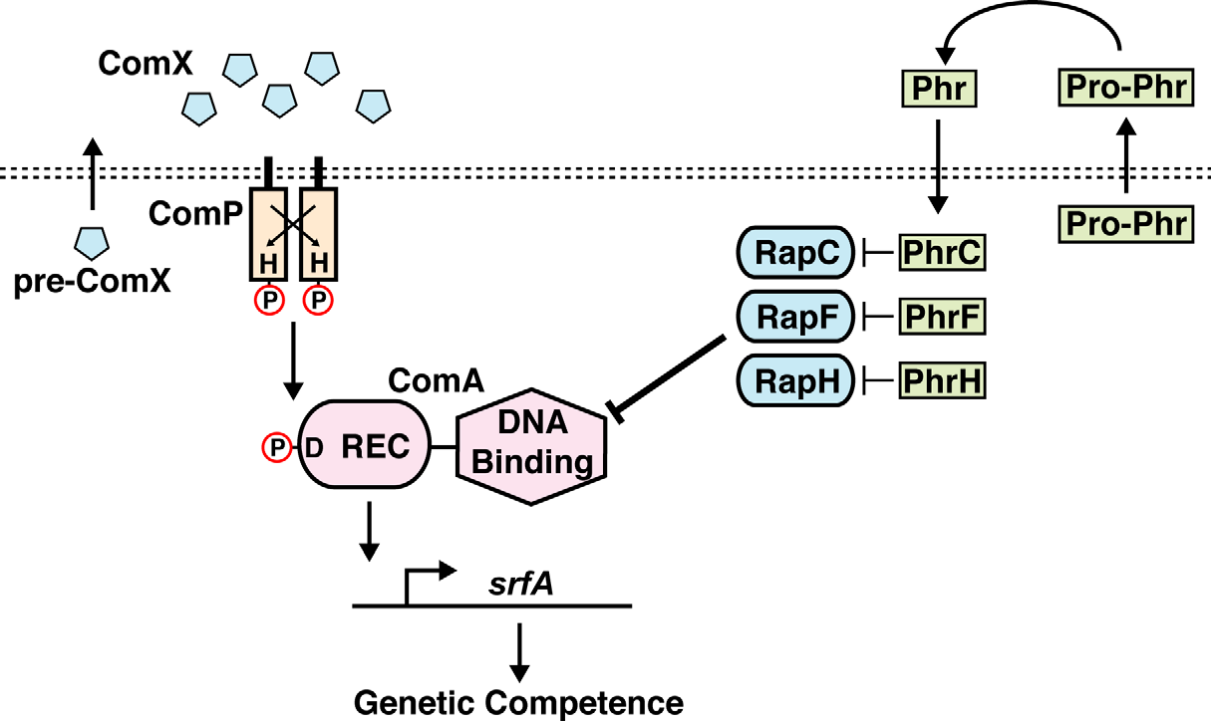
The *B. subtilis* competence signal transduction pathway. ComP autophosphorylates in response to binding the quorum-sensing signal ComX and subsequently phosphorylates ComA. ComA∼P drives transcription of the *srfA* operon that in turn triggers the expression of late-stage competence genes. RapC, RapF, and RapH inhibit the binding of ComA to its target promoters, repressing the induction of genetic competence. Mature Phr peptides are generated proteolytically from pro-Phr proteins during export. The Spo0K permease (not pictured) imports Phr peptides into the cytoplasm where they antagonize Rap protein function. H, histidine; D, aspartic acid; P, phosphoryl group.

*B. subtilis* encodes 11 Rap proteins on its chromosome and another five on plasmids [10]–[12]. Rap proteins were named after the founding members of the family, which were shown to be *r*esponse regulator *a*spartate *p*hosphatases [13]. Rap proteins including RapA, RapB, RapE, RapH, and RapJ are phosphatases that dephosphorylate the intermediate response regulator Spo0F, which consists solely of a REC domain [5],[13]–[15]. In contrast, genetic and biochemical results show that a subset of Rap proteins, including RapC, RapF, and RapH, are anti-activators that bind to the response regulator transcription factor ComA, inhibiting its interaction with DNA promoters without affecting its phosphorylation state (Figure 1) [2],[3],[5]. Additional genetic results also suggest that RapD, RapG, and RapK may inhibit ComA as well [16]–[18]. Similarly, *B. subtilis* RapG inhibits the binding of the response regulator transcription factor DegU to its target promoters [4]. Consistent with the inability of the Rap proteins to dephosphorylate ComA or DegU, it was previously shown that RapC, RapF, and RapH interact stably with the ComA helix-turn-helix (HTH) DNA binding domain and not with its REC domain [2],[5].

Secreted signals called Phr peptides contribute to the complexity of Rap protein signaling. Phr peptides are imported into the cell, where they bind to Rap proteins and antagonize their activity (Figure 1) [19],[20]. Mature Phr peptides are generated from immature pro-Phr polypeptides [21],[22]. The genes encoding the pro-Phr polypeptides overlap with the 3′ end of the *rap* genes, forming *rap-phr* gene cassettes. Pro-Phr polypeptides are secreted from the cell and subsequently undergo proteolytic maturation. Mature Phr peptides are then imported into the cell, where each peptide inhibits its cognate Rap protein (e.g., PhrA inhibits RapA, and PhrC inhibits RapC) and in some cases a non-cognate Rap protein [23]–[25].

To demonstrate how anti-activators such as the Rap proteins inhibit the binding of response regulators to target DNA promoters, we determined the X-ray crystal structure of a *B. subtilis* Rap protein, RapF, in complex with the DNA binding domain of the response regulator ComA (ComA_C_). As expected, comparison of the RapF-ComA_C_ crystal structure with the previously determined structure of RapH-Spo0F [15] revealed that RapF and RapH are structurally similar; however, ComA_C_ and Spo0F bind to RapF and RapH, respectively, at distinct, non-overlapping sites. Furthermore, we show that RapF is monomeric either alone or in complex with PhrF, and that RapF undergoes a conformational change upon binding PhrF that is likely the cause of ComA dissociation from RapF-ComA complexes. Finally, we propose a model that explains the long-standing observation that some Rap proteins dephosphorylate response regulator REC domains while others sequester response regulator HTH DNA binding domains.

## Results

### Overall Structure of the RapF-ComA_C_ Complex

**Figure 2:**
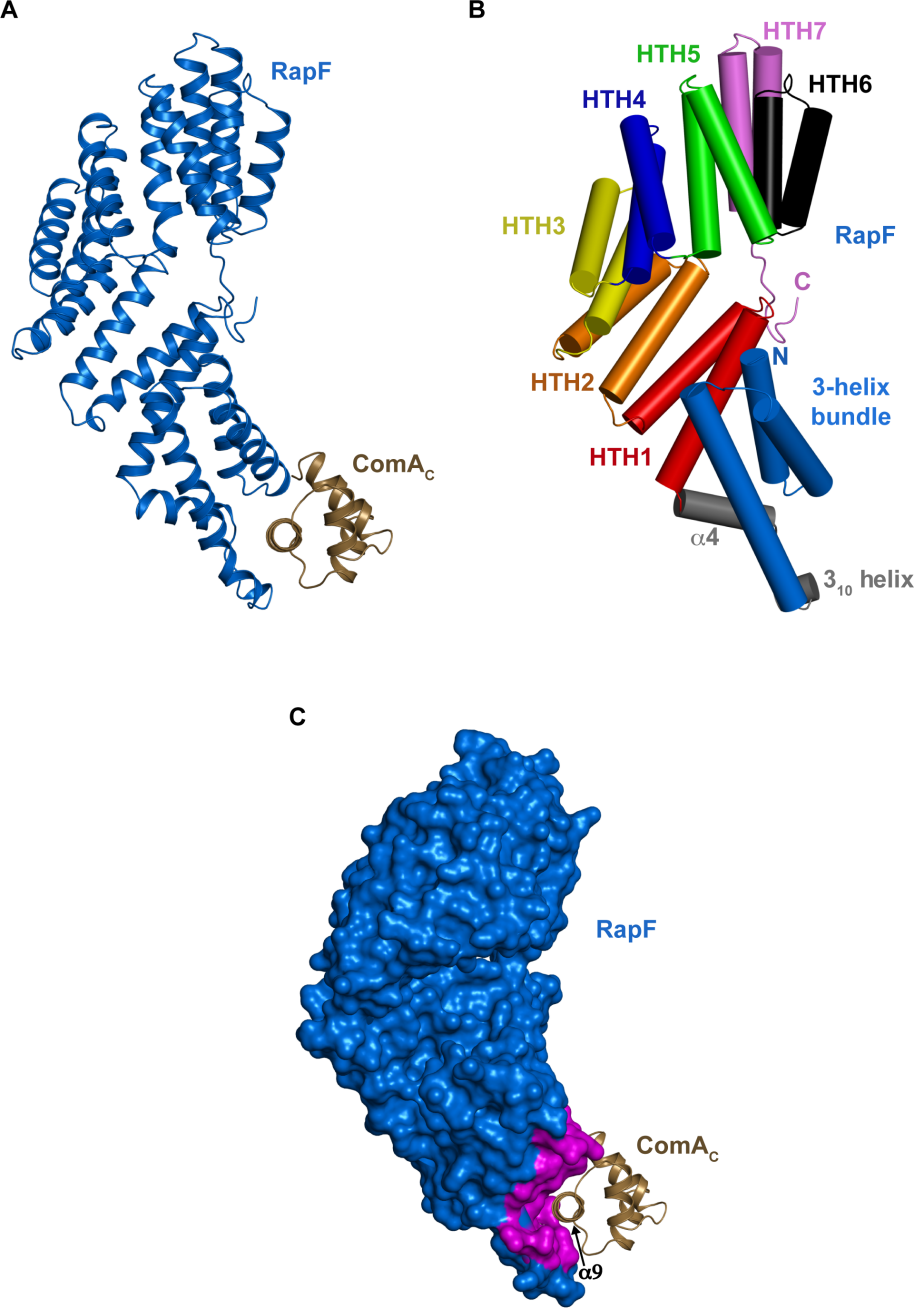
RapF-ComA_C_ structure. (A) The RapF-ComA_C_ crystallographic asymmetric unit. (B) RapF domain architecture with α-helices depicted as cylinders. HTH, helix-turn-helix. (C) RapF-ComA_C_ oriented as in panel B and looking down the helical axis of the principal ComA DNA binding helix α9. RapF residues buried in the RapF-ComA_C_ interface are colored magenta.

To begin to determine how RapF inhibits response regulator binding to DNA without affecting its phosphorylation state, we solved its X-ray crystal structure in complex with ComA_C_ to 2.30 Å resolution (Figure 2A and Table S1). The crystallographic asymmetric unit contains one molecule of RapF bound to one molecule of ComA_C_. It is worth noting that the RapF-ComA_C_ crystallization conditions are identical to conditions that yield crystals in our initial screens containing RapF and full-length ComA (ComA_FL_). However, RapF-ComA_FL_ crystals diffracted anisotropically and to very low resolution. Presumably, the ComA N-terminal REC domain, which does not interact with RapF [2], is responsible for the disorder observed in the RapF-ComA_FL_ crystals.

### RapF Structure

**Figure 3:**
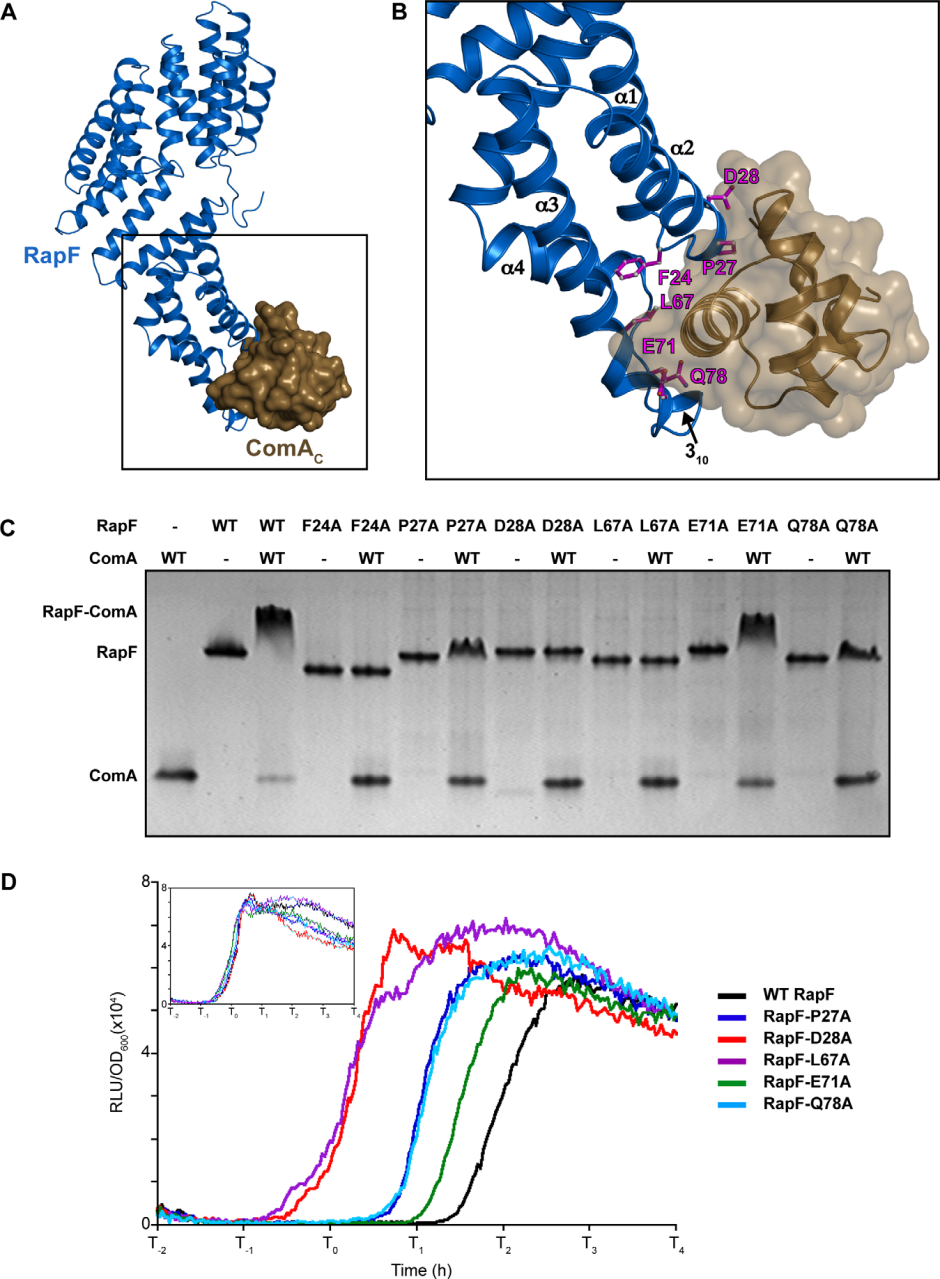
In vitro and in vivo activity of RapF mutants targeting the RapF-ComA interface. (A) RapF (blue cartoon) in complex with ComA_C_ (brown surface). (B) Expanded view of the area enclosed by the black square in panel A. RapF residues targeted for in vitro or in vivo functional analysis are depicted as magenta sticks. (C) Native PAGE analysis of RapF-ComA complexes. (D) In vivo activity of RapF and RapF mutants targeting the RapF-ComA interface measured as a function of P*srfA*-*luc*. The inset panel shows that the P*srfA*-*luc* strains exhibited similar ComA activity in the absence of induced RapF. Each curve is representative of at least three independent experiments performed in duplicate. T_0_ is the time of transition from exponential growth to the stationary phase. Western blotting showed that RapF was overexpressed from the P*spank(hy)* promoter at 2.5 times the level of endogenously produced RapF at T_0_ (unpublished data). RLU, Relative Luminescence Units.

RapF consists of two distinct domains, an N-terminal antiparallel 3-helix bundle (residues 7–72) and a C-terminal tetratricopeptide repeat (TPR) domain (residues 92–381) (Figure 2B). The RapF N-terminal 3-helix bundle consists of two similarly sized helices, α1 (residues 8–23) and α2 (residues 26–42), and a significantly longer third helix, α3 (residues 47–72). The 3-helix bundle and TPR domain are connected by an elaborate linker region (residues 73–91), containing a 3_10_ helix (residues 75–77) joined by a short loop (residues 78–80) to helix α4 (residues 81–91). As discussed in detail below, the N-terminal 3-helix bundle and the linker region form the ComA_C_ binding surface (Figures 2C, 3A, and 3B). The RapF C-terminal TPR domain contains seven helix-turn-helix (HTH) folds that assemble into a large superhelix. HTH folds 1–5 and 7 are bona fide TPR folds containing TPR signature sequences (Figure 2B) [26]. The sixth HTH fold lacks the TPR signature motif but perpetuates the TPR superhelix. Therefore, we refer to the entire C-terminal region containing seven tandem HTH folds as the TPR domain.

### RapF-ComA_C_ Interface

**Figure 4:**
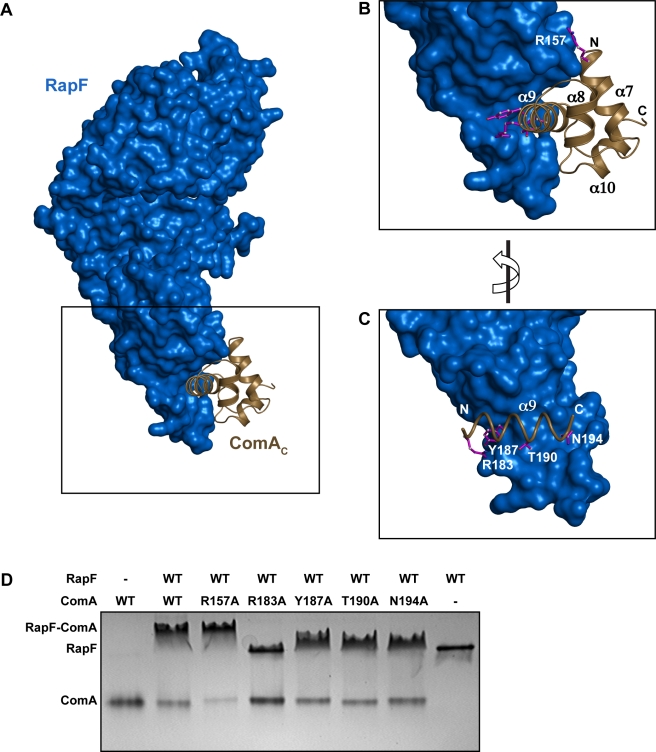
In vitro binding of wild-type RapF to His-ComA mutants targeting the RapF-ComA interface. (A) RapF (blue surface) in complex with ComA_C_ (brown cartoon). (B) Expanded view of the area enclosed by the black rectangle in panel A. (C) RapF interaction with the principal ComA DNA binding helix α9 (brown helix). (D) The binding of wild-type RapF to His-ComA mutants corresponding to the residues depicted as magenta sticks in panels B and C was determined by native PAGE.

The RapF-ComA_C_ X-ray crystal structure reveals an extensive protein-protein interface that buries a total of 1,845.7 Å^2^ surface area (Figures 3A and 4A). The structure of ComA_C_ and its interaction with target binding sites within the *srfA* promoter were previously studied using NMR spectroscopy [27]. The RapF-ComA_C_ crystal structure shows that most of the ComA residues that interact with the DNA phosphate backbone, including Ser181, Ser184, Tyr187, Ser188, and Thr190, are buried in the RapF-ComA_C_ interface (Figures 4B, 4C, and S1 and unpublished data). Furthermore, both of the ComA residues shown to interact with the DNA major groove, specifically Arg183 and Tyr187, are buried in the RapF interface (Figures 4C, S1, and S2).

In sum, the RapF-ComA_C_ interaction buries six of the seven ComA DNA binding residues. It is also notable that in addition to interacting with ComA DNA binding residues, RapF mediates extensive interactions with ComA_C_ residues not directly involved in DNA binding. As described in detail below, we rigorously tested the functional importance of the crystallographically observed RapF-ComA_C_ interface both in vitro and in vivo.

### Functional Analysis of the RapF-ComA Interface In Vitro

To begin to test the functional significance of the RapF-ComA interactions observed in the RapF-ComA_C_ crystal structure, we generated RapF mutants containing individual alanine substitutions at RapF-ComA interfacial positions (Figures 3B and S1), and native PAGE was used to examine the binding of the RapF mutants to wild-type full-length ComA (Figure 3C). RapF Asp28 hydrogen bonds to the ComA Arg157 and Thr155 side chains, and RapF Leu67 forms a hydrophobic interaction with ComA Tyr187 (Figure S1). Consistent with these observations, RapF-D28A and RapF-L67A exhibited a complete loss of ComA binding activity (Figure 3C). Furthermore, RapF P27A forms a hydrophobic interaction with ComA Ile161 and Ser188, and RapF Gln78 hydrogen bonds with ComA Asn194 and with a water molecule at the RapF-ComA interface (Figure S1). Compared to the interaction of wild-type RapF with ComA, the interaction of RapF-P27A and RapF-Q78A with ComA was significantly reduced (Figure 3C).

In addition to the RapF mutants evaluated above, we analyzed the binding of RapF-F24A to wild-type ComA (Figure 3C). RapF Phe24 is located in the loop connecting RapF helices α1 and α2 (Figure 3B). Its main-chain carbonyl oxygen forms a hydrogen bond with the ComA Ser191 side-chain hydroxyl, and its carbonyl carbon atom mediates hydrophobic interactions with ComA Lys195 (Figure S1). Native gel analysis showed that the F24A mutation in RapF results in a complete loss of ComA binding (Figure 3C). While a portion of the RapF Phe24 side chain and main chain are buried in the ComA interface, the rest of its side chain is buried in a hydrophobic pocket formed by residues in RapF helices α3 and α4. We propose that the interaction of the RapF Phe24 side chain with RapF helices α3 and α4 is essential for the RapF-ComA interaction because it (1) positions the RapF Phe24 main chain carbonyl for interaction with ComA and (2) mediates the packing of the RapF 3-helix bundle and linker, which both in turn interact with ComA. Also consistent with this hypothesis, Phe24 is conserved in the Rap proteins previously shown to bind ComA directly [2],[3],[5].

One RapF mutant, RapF-E71A, exhibited a significant but subtler ComA-binding defect than the other RapF mutants tested (Figures 3C and unpublished data). While RapF Glu71 mediates contacts with ComA_C_ (Figure S1), it is located at the periphery of the RapF-ComA interface where it is exposed to bulk solvent. Consistent with previous studies of protein-protein interfaces (see, for example, [28]), interfacial residues such as RapF Glu71 that are exposed to bulk solvent routinely have little impact on the free energy of binding.

In addition to the RapF mutants described above, we engineered full-length ComA mutants containing single alanine substitutions at the RapF-ComA_C_ interface (Figures 4A–C and S2). The binding of the ComA mutants was then evaluated using native PAGE (Figure 4D). ComA Arg183 forms a salt bridge with RapF Glu71 (Figures S1 and S2), and it also interacts intramolecularly with Tyr187, which is also in the RapF interface. ComA-R183A displayed a nearly complete loss of RapF binding, while numerous other ComA mutants, including ComA-Y187A, ComA-T190A, and ComA-N194A, displayed significantly reduced RapF binding activity compared to wild-type ComA (Figures 4D and S2). Only one mutant tested, ComA-R157A, appeared to bind RapF with a similar affinity to wild-type ComA (Figure 4D). As described above, ComA Arg157 and ComA Thr155 hydrogen bond to RapF Asp28 (Figure S1). ComA-T155A was insoluble and therefore could not be studied in vitro, but it is likely that the interaction of ComA Thr155 and RapF Asp28 is primarily responsible for the complete loss-of-function displayed by RapF-D28A (Figure 3C). Finally, we estimate based on the results of the qualitative analysis (Figures 3C and 4D) and additional native PAGE binding studies employing a broad concentration range of RapF and a fixed quantity of ComA (unpublished data) that the dissociation constant (K_d_) for RapF-ComA complex formation is in the 1–10 µM range.

### In Vivo Functional Analysis of the RapF-ComA Interface

To confirm that the RapF-ComA interface observed in the crystal structure is important for the regulation of ComA activity in vivo, we overexpressed RapF mutants containing single alanine substitutions in RapF-ComA interfacial positions and evaluated their affects on ComA transcriptional activity in *B. subtilis* (Figure 3D). More specifically, we measured RapF activity as a function of ComA-driven *srfA* transcription using the *Photinus pyralis* luciferase gene fused to the *srfA* promoter. As previously described, *P. pyralis* luciferase activity corresponds closely with the rate of its transcription in *B. subtilis* [15],[29].

The P*srfA*-luc reporter bioassay shows that *srfA* expression is delayed in proportion to the amount of RapF induced (Figure S3A). However, we questioned why *srfA* was expressed at all considering the fact that the cells overexpressed RapF, which inhibits ComA-driven *srfA* expression. We hypothesized that PhrF expressed from its endogenous locus (Figure S4) was antagonizing RapF activity. To confirm that this was the case, we overexpressed a RapF mutant, RapF-D194N, which contains a mutation previously shown to render RapA and RapC immune to the effects of their cognate Phr peptides [3],[13]. Indeed, overexpressed RapF-D194N completely suppressed P*srfA*-luc expression throughout the course of the entire experiment (unpublished data). Moreover, when we added synthetic PhrF peptide to the P*srfA-luc* reporter strain overexpressing RapF, P*srfA*-luc expression was indistinguishable from the non-induced control (Figure S3B). Together, these results show that overexpressed RapF is antagonized at least to some degree by PhrF expressed from its endogenous locus. Nonetheless, overexpressing RapF significantly delays *srfA*-luc expression. Consistent with these results and as described below, strains overexpressing RapF proteins containing mutations in the RapF-ComA interface should express *srfA*-luc at earlier time points than the wild-type RapF control.

Mirroring the results of the RapF-ComA binding studies, RapF-D28A and RapF-L67A displayed a loss-of-function, and RapF-P27A and RapF-Q78A displayed intermediate phenotypes (Figure 3D). Also consistent with the in vitro results (Figures 3C), RapF-E71A displays a slight loss of function in vivo (Figure 3D). Western blotting confirmed that the RapF mutants were produced at levels comparable to wild-type RapF (unpublished data). Thus, the results of the RapF-ComA in vitro binding studies and the RapF-ComA luciferase bioassays show that the RapF-ComA interface identified in the RapF-ComA_C_ crystal structure is functionally important in vitro and in vivo.

### RapF-ComA Stoichiometry

**Figure 5:**
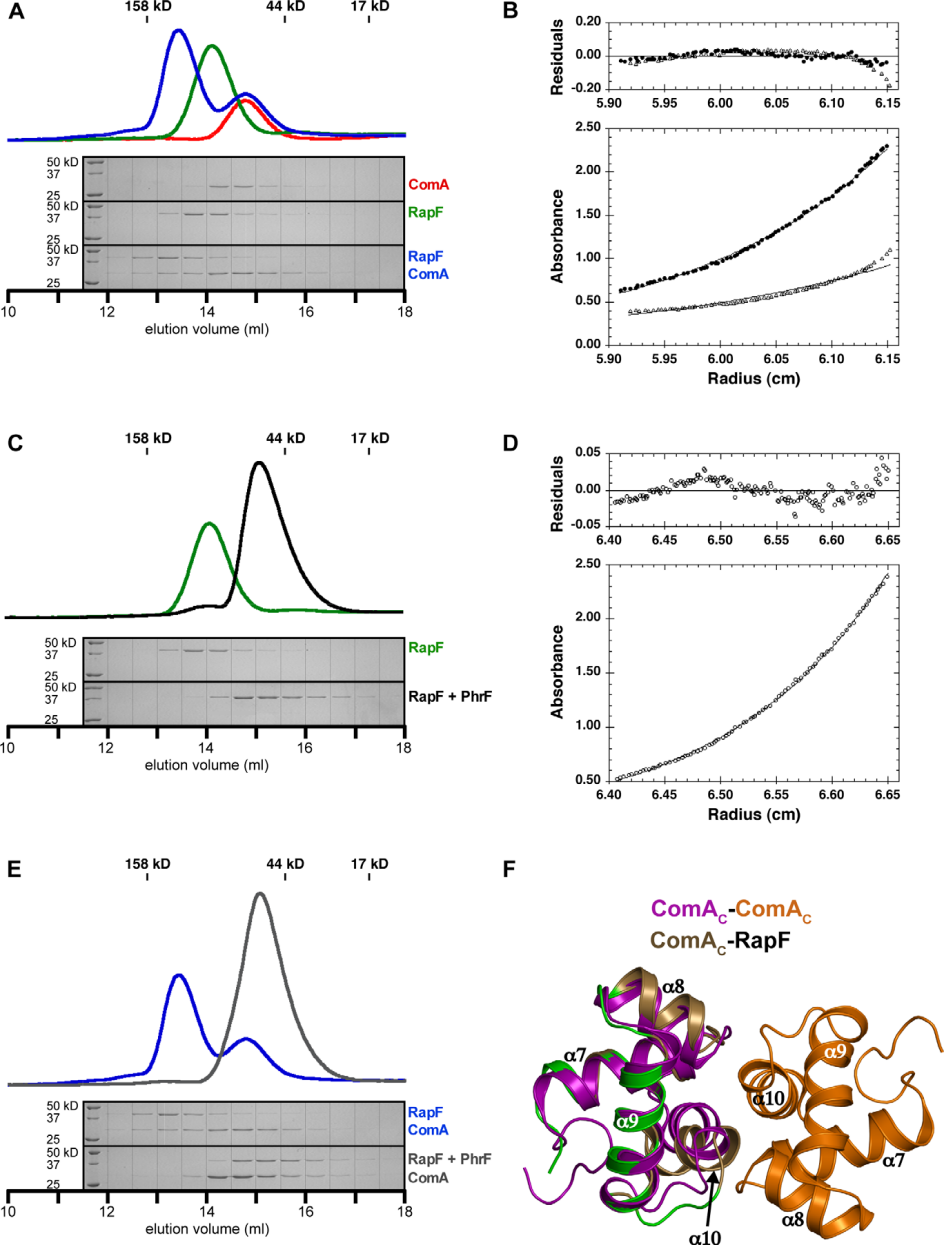
Analysis of RapF complexes in solution. (A) Size exclusion chromatography of RapF (peak elution volume (*V*_R_) 14.11 ml), ComA_2_ (*V*_R_ 14.75 ml), and RapF-ComA (RapF-ComA *V*_R_ 13.43 ml, uncomplexed ComA *V*_R_ 14.80 ml). SDS-PAGE analyses of the indicated fractions are shown below the traces. The peak positions of gel filtration standards are indicated by vertical lines above the traces. (B) Sedimentation equilibrium data for 50 µM RapF (filled circles) and 50 µM RapF-ComA (open triangles). Centrifugation was carried out at 13,000 rpm (RapF) or 9,000 RPM (RapF-ComA) and 20°C as described in the Materials and Methods. Bottom, the measured absorbance at 286 nm (RapF) or 285 nm (RapF-ComA) versus the radius (distance to the center of the rotor) is shown. The continuous line represents the result from a single exponential fit of the data points. Top, the residuals to the fit expressed as the difference between experimental and fitted values. The residuals for the RapF-ComA sample deviated from zero in a systematic fashion, suggesting the presence of non-specific interactions probably between weakly associating ComA dimers in buffer A (see Materials and Methods). (C) Size exclusion chromatography of RapF as in panel A (*V*_R_ 14.11 ml) and RapF+PhrF (*V*_R_ 15.12 ml). SDS-PAGE analysis and peak positions of gel filtration standards are depicted as in panel A. (D) Sedimentation equilibrium data for 50 µM RapF (open circles) mixed with 500 µM PhrF. Top and bottom graphs are depicted as in panel B. Centrifugation was carried out at 13,000 rpm and 20°C as described in Materials and Methods. The absorbance was measured at 286 nm. (E) Size exclusion chromatography of RapF-ComA as in panel A (RapF-ComA *V*_R_ 13.43 ml and uncomplexed ComA_2_ *V*_R_ 14.80 ml) and RapF-ComA+PhrF (*V*_R_ 15.06 ml). Due to their similar size, the RapF-PhrF and ComA_2_ peaks overlap. SDS-PAGE analysis and gel filtration standards are depicted similarly as in panel A. See Table 1 for the molecular weights calculated from the SEC and SE AUC shown in panels A–E. (F) Structural alignment of ComA_C_ from the RapF-ComA_C_ X-ray crystal structure with a molecule of the ComA_C_ homodimer (2KRF) [27]. The ComA_C_ backbone atoms aligned with a root mean-square deviation of 1.40 Å. The dimerization interface consists primarily of residues in helix α10 and the α7–α8 loop. ComA_C_ residues buried in the RapF-ComA_C_ interface are colored green. For clarity, RapF is omitted.

RapF dimerizes around a crystallographic 2-fold symmetry axis in the RapF-ComA_C_ crystals. To begin to determine whether RapF also dimerizes in solution, we analyzed RapF and RapF-ComA complexes by gel filtration (Figure 5A). The results of these studies suggested that RapF alone and RapF in complex with ComA could be forming dimers in solution (Table 1). However, molecular weight determination using gel filtration becomes less reliable as the shape of the analyzed protein or protein complex deviates from that of a sphere, and RapF has an elongated structure (Figure 2A). In fact, RapF is approximately twice as long as it is wide, and its shape more closely resembles a rod than a sphere. Therefore, to determine unambiguously whether RapF dimerizes in solution, we studied RapF using sedimentation-equilibrium (SE) analytical ultracentrifugation (AUC). The theoretical molecular weight of a RapF monomer is 46.3 kD, and its molecular weight as determined by SE AUC is 47 kD (Figure 5B and Table 1). Therefore, we conclude that RapF is monomeric in solution.

**Table 1:** Molecular weight (M) of ComA, RapF, RapF-ComA, and RapF-PhrF complexes determined by size exclusion chromatography (SEC) and analytical ultracentrifugation (AUC).

Sample ID	M_SEC_ (kD)^a^	M_AUC_ (kD)^b^	Monomer M_theoretical_ (kD)	Dimer M_theoretical_ (kD)
ComA	59.1±0.9	N.D.	26.3	52.6
RapF	90.0±1.3	47	45.7	91.4
RapF-ComA	139.7±4.9	74	72.0	144.0
RapF+PhrF	46.7±0.9	50	46.3	92.6
RapF-ComA+PhrF	49.0±0.3	N.D.	46.3 (RapF+PhrF), 26.3 (ComA)	92.6 (RapF+PhrF), 52.6 (ComA)

a The SEC data represent an average of measurements obtained from two independent experiments and were determined as described in Materials and Methods.

b The AUC data represent the molecular weight determined from the linear fit applied to the ln absorbance (285 nm or 286 nm, as indicated) versus the radius squared (not shown) of the data presented in Figure 5B and 5D. N.D, not determined.

The RapF-ComA_C_ crystallographic asymmetric unit (Figure 2A) contains one molecule of RapF bound to one molecule of ComA_C_, suggesting that perhaps a monomer of RapF binds to a monomer of ComA in solution. However, ComA alone and ComA_C_ alone homodimerize in solution (Figure 5A, Table 1) [27],[30], and ComA dimers bind to DNA regulatory sites composed of three recognition elements [30]. Therefore, we examined whether RapF is binding a ComA monomer or dimer in solution using gel filtration in combination with total amino acid analysis and SE AUC. A mixture of RapF and ComA was passed over a gel filtration column and total amino acid analysis was performed on the RapF-ComA complex fractions as described in Materials and Methods. The results of the total amino acid analysis (unpublished data) indicate that the stoichiometry of the RapF-ComA complex is 1∶1. Finally, SE AUC analysis of the identical RapF-ComA sample indicated the formation of a single species of approximately 74 kD (Figure 5B). This is consistent with a complex composed of one molecule of RapF (45.7 kD) and one molecule of ComA (26.3 kD) (Table 1).

Together, the results of the SE AUC, gel filtration chromatography combined with total amino acid analysis, and X-ray crystallography suggest that a monomer of RapF binds to a monomer of ComA or ComA_C_, and that the stoichiometries of the RapF-ComA and RapF-ComA_C_ complexes are 1∶1. In Figure 5F, ComA_C_ from the RapF-ComA_C_ X-ray crystal structure is aligned with a molecule of ComA_C_ from the dimeric ComA_C_ NMR structure [27]. While the RapF-ComA_C_ structure shows that RapF-bound ComA_C_ undergoes conformational changes near the ComA_C_ dimerization interface, we cannot rule out the possibility that these changes were influenced by lattice contacts involving ComA_C_. Nonetheless, consistent with the RapF-ComA_C_ crystal structure, we find that RapF binds to a ComA monomer in solution. Furthermore, while RapF does not interact with ComA_C_ dimerization interface residues, which are located in helix α8, α10, and the α7–α8 loop (Figure 5F), we hypothesize that the RapF-induced ComA_C_ conformational change is transmitted to ComA α10 through contacts between RapF and ComA residues in the C-terminus of α9 (residues N194 and K195) and the α9–α10 loop (residues N197, V198, and G199) (Figure 5F). In fact, RapF Gln78 and RapF Phe24 contact ComA Asn194 and Lys195, respectively, and RapF-Q78A and RapF-F24A both exhibited severe ComA binding defects (Figure 3C). Despite the numerous contacts between RapF and ComA residues directly adjacent to the ComA_C_ dimerization interface, because RapF does not interact with the surface of the ComA_C_ dimerization interface, we conclude that RapF allosterically inhibits ComA_C_ dimerization.

### RapF Undergoes a Conformational Change Upon Binding to PhrF

The gel filtration analysis of RapF-ComA and RapF performed above (Figure 5A) were repeated in the presence of PhrF (Figure 5C, 5E and Table 1). Consistent with previous native-PAGE studies [2], we found that PhrF caused the dissociation of the RapF-ComA complex (Figure 5E and Table 1). Also consistent with these native-PAGE studies where a faster migrating RapF band was observed in the presence of PhrF, we found that PhrF caused RapF to elute at a significantly higher volume than RapF alone (Figure 5C, 5E, and Table 1). However, in the previous native PAGE studies it was assumed that RapF was dimeric [2], and why a faster migrating band appeared in the presence of PhrF was unknown.

As discussed above, SE AUC shows that RapF alone is monomeric (Figure 5B and Table 1). Consistent with this result, the molecular weight of RapF complexed with PhrF is 50 kD as determined by SE AUC (Figure 5D and Table 1), and there is no change in RapF stoichiometry upon binding to PhrF. Therefore, we conclude that RapF migrates through the gel filtration matrix radically slower in the presence of PhrF than in its absence, and RapF migrates faster in the presence of PhrF when analyzed by native PAGE [2], because it undergoes a large conformational change upon binding to PhrF.

## Discussion

Response regulators are the primary mechanism for converting sensory perception into transcriptional output in bacteria. How auxiliary factors such as the Rap proteins function structurally to inhibit response regulator binding to DNA promoters was not known. The X-ray crystallographic, biochemical, and genetic results presented here reveal that RapF disrupts response regulator binding to DNA using a two-pronged mechanism. First, RapF buries ComA DNA binding residues in the RapF-ComA interface, sterically blocking the binding of ComA to its DNA recognition elements. Second, RapF binding allosterically weakens the ComA_C_ dimerization interface, inhibiting the formation of transcriptionally active ComA dimers (Figure 5B). In theory, each mechanism alone could effectively inhibit response regulator activity; however, RapF uses both.

To our knowledge, the data presented here reveal the first atomic level insight into the inhibition of response regulator DNA binding by an anti-activator. However, the structures or regulatory mechanisms of other anti-activators and anti-repressors that target transcription factors other than response regulators have been described, for example TraM, CarS, TrlR, AppA, and ExsD (Figure S5) [31]–[35]. These proteins repress the activity of their targets by (1) directly blocking the interaction of DNA binding residues and DNA, (2) inhibiting their functionally required multimerization, or (3) causing allosteric rearrangements that preclude their binding to DNA. RapF does not share obvious structural similarities with these anti-activators and anti-repressors (Figure S5); however, a comparison of the structural and functional data available for the different systems is informative.

The TraR-TraM crystal structure showed that the anti-activator TraM interacts with the TraR N-terminal dimerization domain (NTD) in addition to its C-terminal DNA binding domain (CTD) and linker region connecting the NTD and CTD (Figure S5) [35]. In contrast to the interactions observed in the RapF-ComA_C_ structure, TraM does not contact the TraM DNA binding residues. Rather, TraM allosterically regulates TraR activity.

CarS-CarA interactions were mapped using NMR chemical shift perturbation and site-directed mutagenesis studies [31]. Interestingly, like RapF (discussed below), the anti-repressor CarS mimics DNA to interact with its target's DNA binding domain; however, the RapF and CarS folds are entirely different (Figure S5). While RapF is entirely alpha helical, CarS resembles an SH3 domain and consists of an antiparallel beta sheet and a 3_10_ helix.

How TrlR, AppA, and ExsD function structurally to regulate their targets is not as well understood as it is for the systems discussed above. However, it was shown that TrlR is a truncated form of TraR that regulates TraM by forming inactive TrlR-TraR dimers [32], and that the flavin binding protein AppA inhibits PpsR repressor activity by reducing a disulphide bond in PpsR and forming inactive AppA-PpsR_2_ complexes [36]. Finally, how ExsD functions structurally to inhibit ExsA transcriptional activity is unknown, and different studies suggest that ExsD interacts stably with either the ExsA N-terminal oligomerization domain alone [37] or only the intact full-length protein [38].

**Figure 6:**
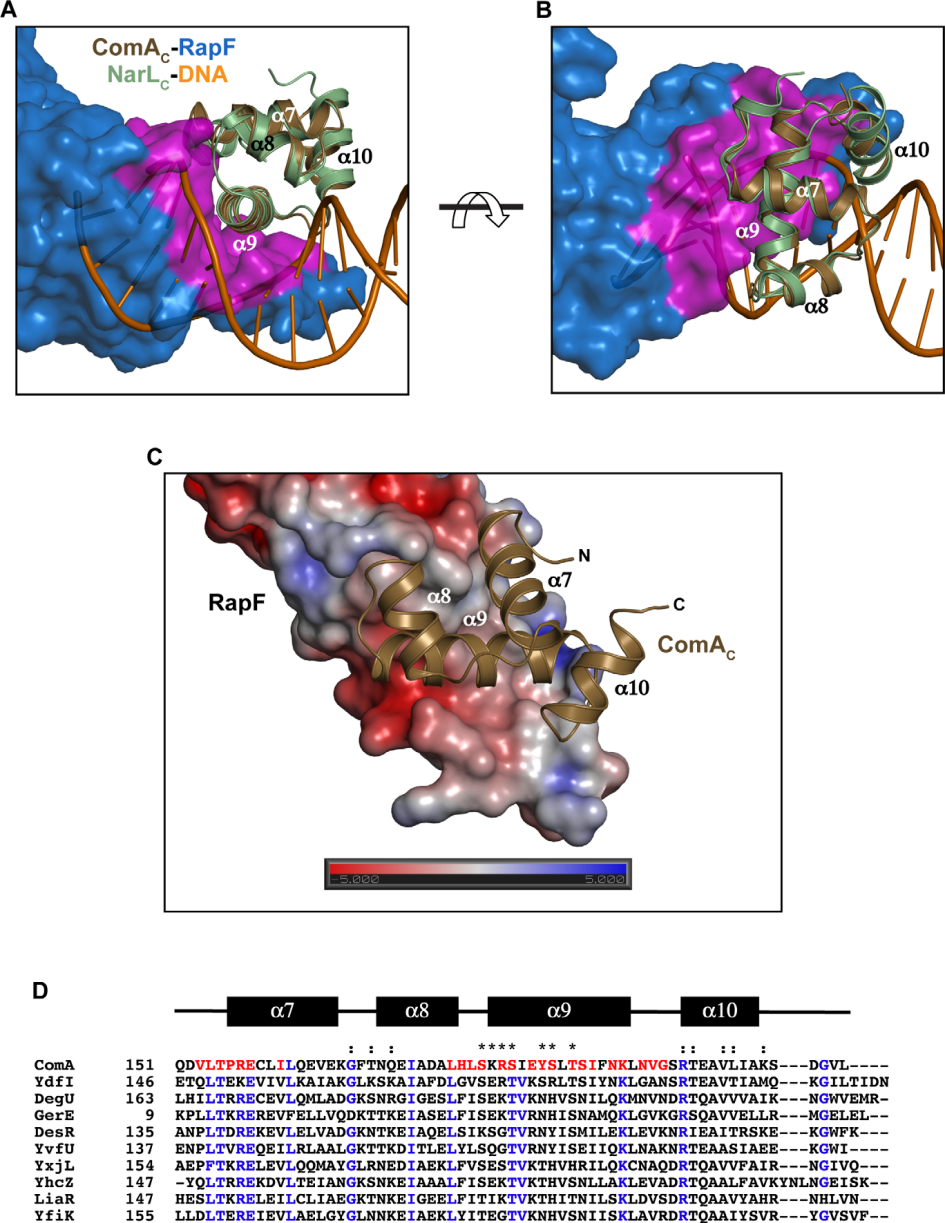
RapF mimics DNA. (A) Structural alignment of ComA_C_ of the RapF-ComA_C_ structure with a molecule of NarL_C_ of the NarL_C_-DNA structure (1JE8) [41] oriented looking down the helical axis of ComA_C_ helix α9 shows that the RapF ComA binding surface (magenta) resembles the shape of the DNA major groove. The ComA_C_ and NarL_C_ Cα backbone atoms aligned with a root mean-square deviation of 0.60 Å. (B) Top view of the alignment shown in panel A obtained by rotating panel A 90° in the direction indicated by the arrow. (C) The RapF electrostatic surface potential was calculated using APBS [43] and displayed on the solvent-accessible surface. Electronegative and electropositive surfaces are colored red and blue, respectively, and contoured from −5 to +5 kT/e. (D) *B. subtilis* LuxR/FixJ/NarL-type family member HTH DNA binding domain amino acid sequence alignment. Highly conserved residues are indicated with blue type. ComA secondary structure assignments are denoted by the black rectangles above the sequences. ComA residues buried in the RapF interface are indicated with red type. Asterisks and colons above the sequences denote ComA DNA binding residues and ComA_C_ dimerization interface residues, respectively [27].

ComA is a member of the LuxR/FixJ/NarL family of transcriptional regulators whose HTH DNA-binding domains are tetra-helical bundles [39],[40]. Aligning ComA_C_ of the RapF-ComA_C_ structure with a molecule of NarL_C_ of the NarL_C_-DNA complex structure (1JE8) revealed that RapF's ComA-binding surface adopts a conformation reminiscent of a DNA major groove (Figure 6A and 6B) [41],[42]. Additionally, analogous to DNA, at the core of the RapF ComA_C_-binding surface, there is a large electronegative patch (Figure 6C) [43]. Moreover, six of the seven previously identified ComA DNA binding residues are buried in the RapF-ComA_C_ interface. For example, the sidechain of ComA Arg183 forms a salt bridge with the side chain of RapF Glu71, and the side chain of ComA Thr190 forms a hydrogen bond with the side chain of RapF Glu71 (Figure S2). These contacts appear to mimic the hydrogen bonds that the ComA residues make with the DNA major groove and phosphate backbone, respectively [27]. Thus, the RapF-ComA_C_ X-ray crystal structure reveals that ComA binds to a RapF surface that, at least in part, mimics DNA.

HTH DNA binding domains are ubiquitous in all three superkingdoms of life [39]. In fact, *B. subtilis* alone encodes 10 proteins containing tetra-helical bundle HTH domains similar to the ComA DNA binding domain [44]. The identity of residues in helix α9 and the α8–α9 loop are primarily responsible for determining LuxR/FixJ/NarL-type HTH DNA recognition element specificity [27],[41],[45],[46]. For example, ComA Tyr187 in helix α9 inserts into the DNA major groove and contacts the phosphate backbone [27], while no other *B. subtilis* response regulator encodes tyrosine at the equivalent position (Figure 6D). Similarly, ComA Arg183 and Thr190 insert into the DNA major groove and contact the phosphate backbone, respectively. Only one other *B. subtilis* response regulator, YdfI, encodes identical residues at these positions (Figure 6D). YdfI is in fact the *B. subtilis* protein containing a LuxR/FixJ/NarL-type HTH domain most highly homologous to ComA_C_, yet they bind to different DNA recognition elements [30],[47]. Interestingly, we found that purified RapF and YdfI did not stably interact (unpublished data). We propose that Rap protein interactions with the HTH DNA binding residues serve to sterically block the binding of response regulators with their DNA recognition elements. Contacts with these DNA binding residues may enable Rap proteins to preferentially recognize their response regulator targets, e.g. ComA or DegU, among the myriad *B. subtilis* HTH DNA binding domains.

**Figure 7:**
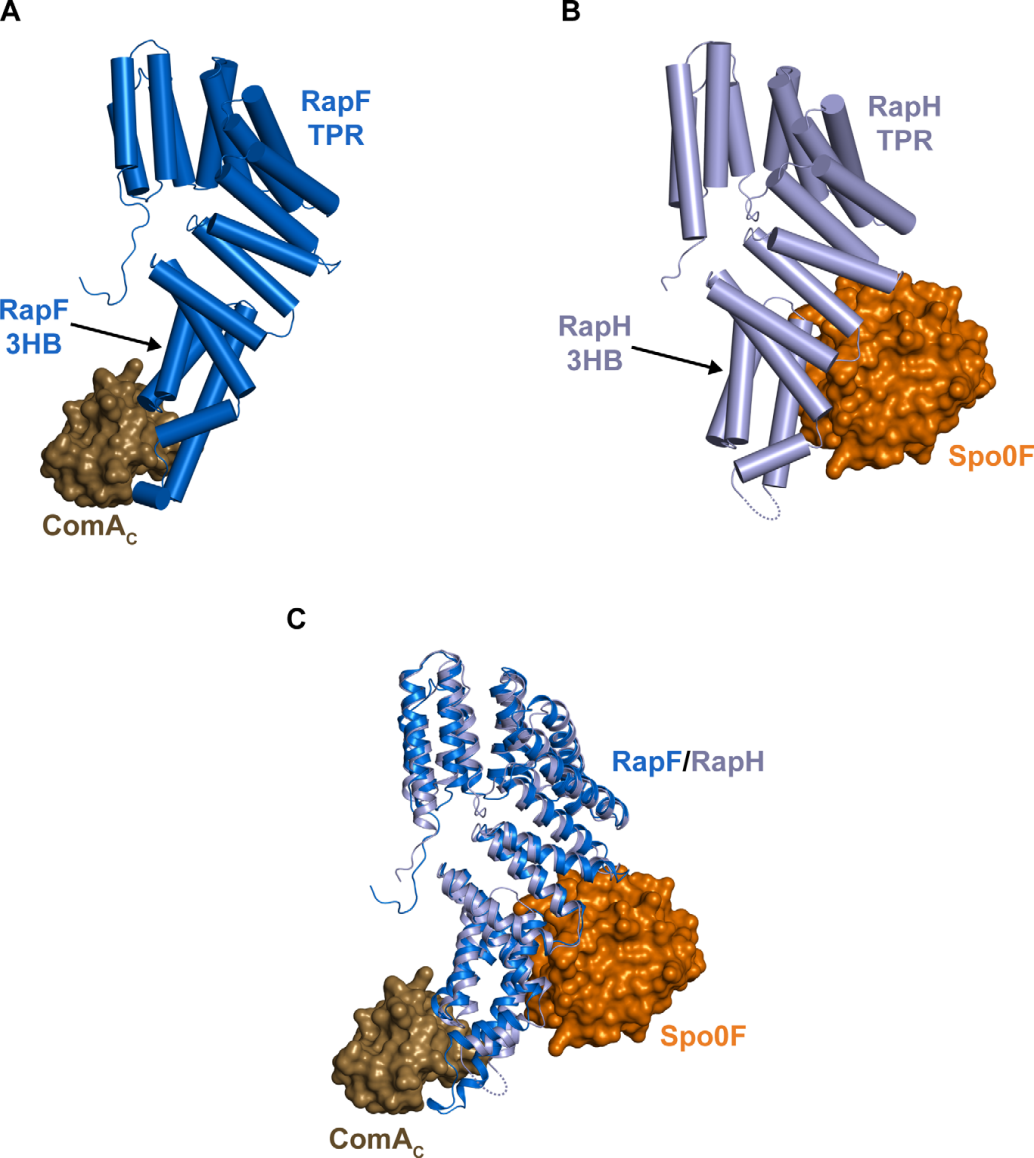
ComA_C_ and Spo0F bind to different Rap protein surfaces. (A) Side view of the RapF-ComA_C_ complex. (B) Side view of the RapH-Spo0F complex (3Q15) [15]. This view was obtained by aligning RapH of the RapH-Spo0F complex with RapF of the RapF-ComA_C_ complex as oriented in panel A and as described in (C). Dashed lines denote the RapH disordered region as described below. A comparison of panels A and B shows that ComA_C_ and Spo0F bind to opposite faces of the RapF and RapH 3-helix bundles, respectively, and that Spo0F also interacts with the RapH TPR domain. (C) Side view of RapF of the RapF-ComA_C_ structure aligned with RapH of the RapH-Spo0F structure. The RapF and RapH Cα backbone atoms aligned with a root mean-square deviation of 1.61 Å. We previously observed insufficient electron density corresponding to RapH residues 69–76, and they were not included in the RapH-Spo0F model [15]. These residues correspond to the C-terminus of RapF helix α3 and a portion of the RapF linker region including the 3_10_ helix. This region appears to be ordered in the RapF structure resulting from extensive interactions with ComA. Structural alignment of RapF of the RapF-ComA_C_ structure with RapH of the RapH-Spo0F structure also revealed conformational differences in the regions surrounding the RapF and RapH α2–α3 loops. This area contains residues particularly important for Rap phosphatase activity, including a catalytic residue that inserts into the Spo0F active site. Thus, the conformational differences between RapF and RapH near the α2–α3 loop likely result from Spo0F binding to RapH.

Some *Bacillus* Rap proteins are phosphatases that bind to and dephosphorylate Spo0F, a stand-alone REC domain, while others bind to the HTH DNA binding domain of the response regulator ComA rather than its REC domain [2],[5],[15]. Structural alignment of RapF of the RapF-ComA_C_ structure with RapH of the RapH-Spo0F structure (3Q15) showed that their tertiary structures are essentially identical, and they align with a root mean-square deviation of 1.61 Å (Figure 7A–C) [15]. Remarkably, this alignment also revealed that ComA_C_ and Spo0F bind to non-overlapping Rap protein surfaces (Figure 7C). ComA_C_ interacts only with the RapF linker region and 3-helix bundle, while Spo0F interacts with the RapH TPR domain and 3-helix bundle at a site distant from the ComA_C_ binding site on the RapF 3-helix bundle.

Rap proteins have not been observed to bind simultaneously to both the REC and HTH DNA binding domains of a single response regulator. However, it is possible that an ancestral Rap protein did in fact interact with both domains. The Spo0F (REC) and ComA_C_ (HTH) binding sites do not overlap, and a Rap protein could theoretically bind to both a REC and HTH domain concurrently (Figure 7C). Alternatively, there may not have been an ancestral Rap protein that could bind a REC and HTH DNA binding domain. In this case, the fact that Rap proteins commonly target one or the other of two domains found in response regulator transcription factors could be a coincidence resulting from independent evolution of Rap protein interactions with their targets.

Furthermore, we speculate that Rap proteins such as RapC, RapF, and RapH may have evolved to interact stably with the ComA HTH domain but not its REC domain [2],[5] because the short linker connecting them precludes their simultaneous interaction with the relevant Rap protein binding sites. Indeed, structural alignment of RapF of the RapF-ComA_C_ structure with RapH of the RapH-Spo0F structure shows that the C-terminus of Spo0F and the N-terminus of ComA_C_ are separated by greater than 49 Å. Rap proteins could interact with the REC and HTH domain of a single response regulator simultaneously if the domains were connected by a long linker as found in the *B. subtilis* uncharacterized protein YesN, or an additional domain common to the response regulators in the NtrC family [40],[44]. However, the relatively short linker region connecting the ComA REC and HTH domains would prevent their simultaneous interaction with the identified Rap protein REC [15] and HTH domain binding sites (Figure 7C).

Previous work suggests that Phr peptides may bind to the Rap protein TPR domain [3],[13],[24], and it was hypothesized that response regulators may compete with Phr peptides for binding to a common site on the TPR domain [3]. However, the RapF-ComA_C_ X-ray crystal structure shows that the RapF TPR domain does not in fact contact ComA_C_. RapF-ComA_C_ contacts are mediated only by the RapF 3-helix bundle and linker region. Thus, it appears that PhrF may allosterically inhibit the RapF-ComA interaction, and the dissociation of RapF and ComA presumably results from the PhrF-induced conformational change in RapF (Figure 5C and 5E). The Rap protein conformational changes induced by Phr peptide binding are currently unknown, and it seems likely that crystal structures of Rap proteins in their Phr peptide bound conformations will be required to reveal these conformational changes as well as the mechanistic nature of the Rap-Phr interaction.

Furthermore, in regard to Rap protein stoichiometry, it is worth noting that RapH dimerizes around a crystallographic 2-fold symmetry axis similar to RapF, and both RapH and RapK migrate through gel filtration matrix in a manner consistent with that of a dimer ([15] and unpublished data). However, SE AUC analysis of both RapH and RapK (unpublished data) show that they are in fact monomeric in solution like RapF. Additional SE AUC studies are required to determine whether other Rap proteins are monomeric similar to RapF, RapH, and RapK, or whether they are dimeric as previously suggested [48].

The results of our X-ray crystallographic studies, SE AUC, and gel filtration combined with total amino acid analysis suggest that a RapF monomer binds to a monomer of ComA or ComA_C_. However, the reported ComA_C_ homodimerization interface residues [27] are not buried in the RapF-ComA interface (Figure 5F). Thus, it is theoretically possible that a molecule of ComA in a RapF-ComA complex could bind to another molecule of ComA using its ComA_C_ homodimerization surface. Also, a molecule of ComA bound to RapF could bind to another molecule of ComA using its N-terminal receiver domain, which might be accessible because it does not interact stably with RapF. However, even if ComA can in fact homodimerize to some extent when bound to RapF, it is in all likelihood a non-functional ComA dimer. That is, if each molecule of ComA in a ComA dimer is required to interact with DNA at a target promoter [30], then RapF would disable the ComA dimer when it binds directly to and buries nearly all of the DNA binding residues belonging to one molecule of ComA.

Rap proteins are proposed to be the most ancient member of the RNPP protein family [49]. The RNPP proteins are related to the *Bacillus R*ap, *N*prR, and *P*lcR proteins, as well as the Enterococcal protein *P*rgX. These proteins are widespread in Firmicutes, and a *rap-phr* cassette was also recently identified outside of the Firmicute phylum encoded on a plasmid in a highly multidrug resistant strain of *Salmonella enterica* serovar Dublin [50]. Structure-function analysis showed that PlcR and PrgX are transcription factors that contain N-terminal DNA binding domains and C-terminal TPR or TPR-like domains [49],[51]. There are no structural data and very limited functional data available describing the NprR proteins, which include a number of proteins in *Bacillus* and *Clostridia*. Like PlcR and PrgX, NprR proteins are transcription factors; however, sequence analysis suggests that NprR proteins are essentially Rap proteins containing a tetrahelical bundle HTH DNA binding domain fused to their N-terminus [20].

We hypothesize that the NprR structure may closely resemble the structure of RapF-ComA_C_, except that in the case of NprR the HTH DNA binding domain is covalently attached to the N-terminus of the NprR 3-helix bundle. Similar to RapF-ComA_C_, DNA binding residues in the NprR HTH DNA binding domain may be buried in an interface with the NprR 3-helix bundle and linker region. Like the Rap proteins, NprR, PlcR, and PrgX activity is regulated by oligopeptides reminiscent of the Phr peptides (review in [52]). Peptide binding to NprR could disrupt the interaction of the NprR 3-helix bundle and its DNA binding domain, triggering NprR transcriptional activity. This mechanism is analogous to the PhrF-driven dissociation of the RapF 3-helix bundle and ComA.

In conclusion, we speculate that a protein containing only the Rap protein 3-helix bundle and linker region may be sufficient to inhibit the binding of HTH DNA binding domains to DNA. Engineering this relatively simple scaffold to bind HTH domains other than those belonging to ComA or DegU would create useful in vivo tools for studying bacterial signaling. Furthermore, Rap-Phr and NprR-Phr systems regulate medically and commercially important phenotypes in numerous *Bacillus* species, e.g. sporulation in *B. anthracis* [53] and sporulation and the production of Cry protein endotoxin in *B. thuringiensis* [52],[54], respectively. Therefore, compounds that regulate the activity of Rap and Rap-like proteins could serve as commercially important additives that modulate the overproduction of insecticidal endotoxins or as antibacterial drugs.

## Materials and Methods

### Protein Purification for Crystallization

RapF was overexpressed as an N-terminal fusion to glutathione S-transferase (GST) in *E. coli* strain BL21 grown in LB media supplemented with 100 µg/ml ampicillin. The cultures were first grown at 37°C to an OD_600_ of 0.4 and then transferred to 25°C and induced with 0.1 mM isopropyl β-D-thiogalactopyranoside (IPTG) at an OD_600_ of 0.6. After growth for an additional 16 h at 25°C, cultures were harvested by centrifugation at 4,000 RPM, lysed in Buffer I (20 mM Tris pH 8.0, 150 mM NaCl, 5 mM DTT) supplemented with 5 mM MgCl_2_, 20 µg/ml DNaseI, and 2 mM phenylmethylsulfonyl fluoride (PMSF), and insoluble material pelleted at 22,000 RPM for 90 min. Cleared lysates were applied to Glutathione-Uniflow Resin (Clontech), equilibrated in Buffer I. The resin was then washed with Buffer I, resuspended in 4 bed volumes of Buffer I and 3.5 µg/ml thrombin, and incubated for 1.5 h at 25°C. This procedure resulted in complete cleavage of the N-terminal GST affinity tag as determined by SDS-PAGE. Following thrombin cleavage, RapF (residues 1–381) contained two heterologous residues (Gly-Ser) derived from the thrombin cleavage signal. RapF was eluted with Buffer I and diluted 2-fold with Buffer II (20 mM Tris pH 8.0, 5 mM DTT), passed through a 0.22 µm filter, and loaded onto an anion exchange column (Source 15Q, GE Healthcare) equilibrated with Buffer II containing 75 mM NaCl. RapF was eluted in a 75–750 mM NaCl linear gradient of Buffer II. RapF-containing fractions were further purified by gel filtration using a Superdex 200 (GE Healthcare) 16/70 column equilibrated with Buffer II containing 150 mM NaCl. Again, RapF containing fractions were pooled and concentrated.

ComA_C_ protein (His-ComA_C_) was purified as described previously with the following modifications to the protocol [2]. Following Ni-NTA affinity column chromatography, His-ComA_C_ containing fractions were pooled, diluted 4-fold with Buffer III (50 mM Tris pH 8.0, 5 mM DTT), passed through a 0.22 µm filter, and loaded onto an anion exchange column (Source 15Q; GE Healthcare) equilibrated with Buffer III containing 50 mM KCl. His-ComA_C_ eluted from the anion exchange column in the flow through and was concentrated by ultrafiltration (MWCO: 3 kDa). For storage, His-ComA_C_ was exchanged into Buffer IV (50 mM 4-(2-Hydroxyethyl)-1-piperazinepropanesulfonic acid (EPPS) pH 8.0, 200 mM KCl, 1 mM DTT) using G-25-50 Sephadex (Sigma).

### Crystallization and Data Collection

RapF-ComA_C_ crystals were produced by the vapor diffusion method at 20°C using a 1∶1 mixture of RapF∶ComA_C_ (100 µM RapF and ComA_C_ each in its corresponding storage buffer) and well solution (6% [w/v] PEG 8,000, 240 mM calcium acetate, 80 mM sodium cacodylate pH 6.5, and 20% glycerol). Data were collected at a wavelength of 1.0750 Å on nitrogen-cooled crystals at NSLS beamline X29A. Diffraction data were processed using HKL2000 [55].

### Structure Determination, Model Building, and Refinement

The RapF-ComA_C_ structure was determined by molecular replacement with PHASER [56] using the N-terminal domain (residues 4–68) and C-terminal domain (residues 77–376) of RapH (3Q15) as search models [15]. ComA_C_ was not included in the search model. The single molecular replacement solution identified using PHASER was refined in REFMAC5 [57]. ARP/wARP was used to generate an initial model [58], and iterative cycles of building in COOT [59] and refinement in PHENIX [60] were performed to complete the model. The final model lacks only residues 1–5 corresponding to the N-terminus of RapF, and 146–152 and 211–214 corresponding to the N- and C-terminus of ComA_C_, respectively. The building of a single Mn^2+^ atom into clear electron density during the final stages of refinement in PHENIX was justified based on its coordination to oxygen ligands, i.e. the carbonyl oxygens of RapF Leu40 and Met43, and the side chain oxygens in the RapF Glu45 side chain, the metal-oxygen ligand distances, the similarity of the Mn^2+^ refined B-factor to those of its surrounding atoms, and the absence of calculated Fo-Fc difference density surrounding the Mn^2+^ ion. Mn^2+^ was not added to the RapF purification or crystallization buffers and may have been acquired through adventitious buffer contamination. Secondary structure assignments were calculated using PROMOTIF [61]. Ramachandran statistics were calculated in Molprobity [62]. Molecular graphics were produced with PyMOL [63]. The RapF-ComA_C_ structure has excellent geometry, with 98.4%, 1.6%, and 0% of residues falling within the favored, allowed, and outlier regions of the Ramachandran plot, respectively.

### Non-Denaturing Polyacrylamide Gel Electrophoresis

10 µM RapF and either 10 µM ComA or 10 µM His-ComA as indicated were incubated in Buffer A (20 mM Tris pH 8.0, 300 mM NaCl, 5 mM MgCl_2_, 1 mM DTT, and 5% glycerol) for 30 min at 4°C (see Text S1 for additional details regarding the purification of ComA and His-ComA). 5× sample buffer was added to each reaction mixture and samples were analyzed by native PAGE using 12% Tris-glycine gels. Gels were run under constant voltage (85 V) for 4 h at 4°C and then stained with Coomassie Brilliant Blue R-250 (Amresco). The disappearance of the lower band (ComA) was the primary indicator of complex formation. The degree of top band (RapF) shifting corresponded with the disappearance of ComA and provided additional evidence of RapF-ComA complex formation.

### Gel Filtration

Gel filtration runs were performed with RapF, His-ComA, and PhrF (NH_2_-QRGMI-COOH) (LifeTein) at 20 µM, 40 µM, and 200 µM, respectively. Prior to analysis, the proteins were incubated in Buffer A for 30 min at 4°C and filtered by centrifugation through a 0.22 µm cut-off cellulose acetate filter (Spin-X; Costar). 100 µl of each sample was injected onto a Superdex 200 HR10/30 gel filtration column (Amersham Biosciences) equilibrated in Buffer A. 0.5 ml fractions were collected and analyzed by SDS-PAGE. Gels were stained with Coomassie Brilliant Blue R-250. RapF-ComA was prepared for total amino acid analysis by injecting a mixture containing 40 µM RapF and 80 µM His-ComA in Buffer A onto a Superdex 200 HR10/30 gel filtration column and collecting fractions spanning 12.0 to 13.5 ml as shown in Figure 5A. The RapF and ComA bands were separated by SDS-PAGE and excised for total amino acid analysis at the Keck Biotechnology Resource Laboratory (Yale).

### Sedimentation Equilibrium Analysis

Analytical ultracentrifugation measurements were carried out on a Beckman XL-A (Beckman Coulter) analytical ultracentrifuge equipped with an An-60 Ti rotor (Beckman Coulter) at 20°C. 50 µM RapF and 50 µM RapF mixed with 500 µM PhrF were dialyzed overnight against 20 mM Tris pH 8.0 and 150 mM NaCl and analyzed at rotor speeds of 13,000 (Figure 5B) and 16,000 rpm (unpublished data). RapF-ComA Superdex 200 HR10/30 gel filtration fractions spanning 12.0 to 13.5 ml as shown in Figure 5A were dialyzed overnight against Buffer A minus DTT and analyzed at rotor speeds of 9,000 rpm (Figure 5D) and 16,000 rpm (unpublished data). Data were acquired at two wavelengths per rotor speed setting and processed simultaneously with a nonlinear least squares fitting routine [64]. Solvent density and protein partial specific volume were calculated according to solvent and protein composition, respectively [65].

### Luciferase Assays

Luciferase assays were performed as previously described with the following modifications [15]. The *PsrfA-luc* reporter strains (see Text S1) were grown in LB medium to an OD_600_ of 2.0, centrifuged, and resuspended in fresh Competence Medium (CM) [66] to an OD_600_ of 2.0. The cultures were then diluted 20-fold in fresh CM supplemented with 0.25 µM IPTG to induce expression of wild-type or mutant *rapF* from the P*spank(hy)* promoter. 200 µl of the induced cultures were dispensed per well in duplicate in a 96-well black plate (Corning). Luciferin was added to each well at a final concentration of 0.47 µM. The cultures were then incubated at 37°C under agitation in a PerkinElmer Envision 2104 Multilabel Reader. The plate lids were heated to 38°C to avoid condensation. Relative Luminescence Unit (RLU) and OD_600_ were measured at 1.78 min intervals.

### Accession Numbers

Atomic coordinates and structure factors for RapF-ComA_C_ have been deposited in the Protein Data Bank under accession code 3ULQ.

## Supporting Information

Figure S1 Schematic representation of the RapF residues targeted for mutagenesis and their interactions at the ComA interface. RapF and ComA residues are depicted with blue and brown bonds, respectively. Hydrogen bonds are depicted as dashed green lines. Blue and brown semicircles with radiating lines depict hydrophobic contacts between RapF and ComA residues, respectively. The schematic was produced with LIGPLOT [67].
(TIF)

Figure S2 Schematic representation of the ComA residues targeted for mutagenesis and their interactions at the RapF interface. ComA and RapF residues are depicted with brown and blue bonds, respectively. Hydrogen bonds are depicted as dashed green lines. Brown and blue semicircles with radiating lines depict hydrophobic contacts between ComA and RapF residues, respectively. The schematic was produced with LIGPLOT [67].
(TIF)

Figure S3 RapF and PhrF regulate the expression of P*srfA*-*luciferase*. (A) P*srfA*-*luc* activity measured in the absence or presence of IPTG at the indicated concentrations. (B) Synthetic PhrF peptide added to the cultures antagonizes the delayed expression of P*srfA*-*luc* caused by the overexpression of RapF.
(TIF)

Figure S4 *B. subtilis* upregulates the expression of *phrF* during the transition to stationary phase growth (T_0_). *PhrF-luc* expression in *B. subtilis* growing in competence media (CM) or sporulation media (DSM). In addition to being driven by a promoter upstream of the *rapF-phrF* operon, *phrF* expression is upregulated during stationary phase by the stationary phase sigma factor, σ^H^, whose binding site lies within *rapF* [68].
(TIF)

Figure S5 The structures of (A) RapF-ComA_C_ (PDB 3ULQ), (B) TraR-TraM (2Q0O) [35], (C) CarS (2KSS) [31], (D) the AppA BLUF domain (2IYG) [33], and (E) ExsD (3FD9) [34]. To our knowledge the TrlR structure has not yet been determined; however, TrlR is a truncated form of TraR (panel B) [32].
(TIF)

Table S1 Data collection and refinement statistics. R_sym_ = Σ_h_ Σ_i_ | I_i_(h)−<I(h)>|/Σ_h_ Σ_i_ I_i_(h), where I_i_(h) is the i^th^ measurement of h and <I(h)> is the mean of all measurements of I(h) for reflection h. R_work_ = Σ ||F_o_|−|F_c_||/Σ |F_o_|, calculated with a working set of reflections. R_free_ is R_work_ calculated with only the test set (5.1%) of reflections. Data for the highest resolution shell are given in parentheses. The structure was determined using a single crystal.
(DOC)

Text S1 Supplemental Materials and Methods.
(DOC)

## Abbreviations

CTD: C-terminal domain
HTH: helix-turn-helix
NTD: N-terminal domain
REC: receiver
SE-AUC: sedimentation-equilibrium analytical ultracentrifugation
TPR: tetratricopeptide repeat
