# Supplementary figures and images for: Structural Basis of Response Regulator Inhibition by a Bacterial Anti-Activator Protein
Source: PLoS Biol. 2011 Dec 27;9(12):e1001226. doi: 10.1371/journal.pbio.1001226 (PMC3246441; doi:10.1371/journal.pbio.1001226)

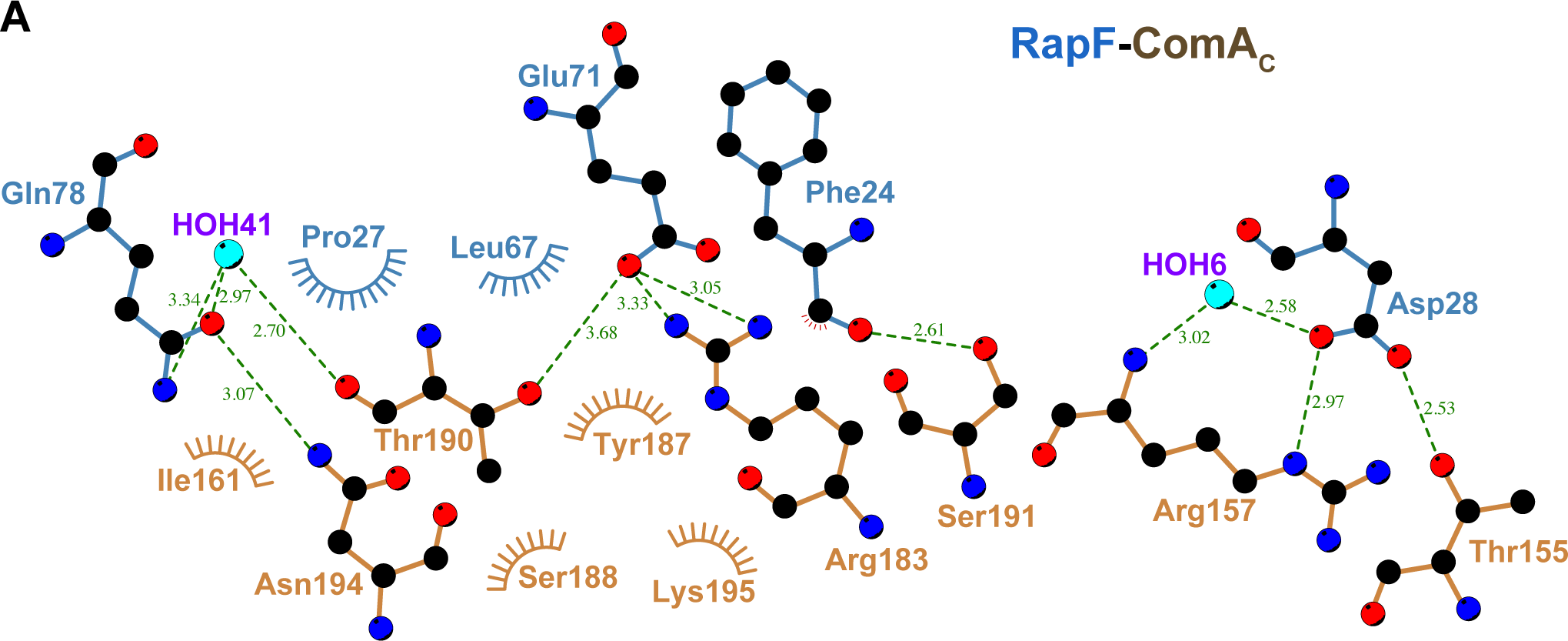

Supplement: Figure S1 — Schematic representation of the RapF residues targeted for mutagenesis and their interactions at the ComA interface. RapF and ComA residues are depicted with blue and brown bonds, respectively. Hydrogen bonds are depicted as dashed green lines. Blue and brown semicircles with radiating lines depict hydrophobic contacts between RapF and ComA residues, respectively. The schematic was produced with LIGPLOT [67]. (TIF) [file pbio.1001226.s001.tif]

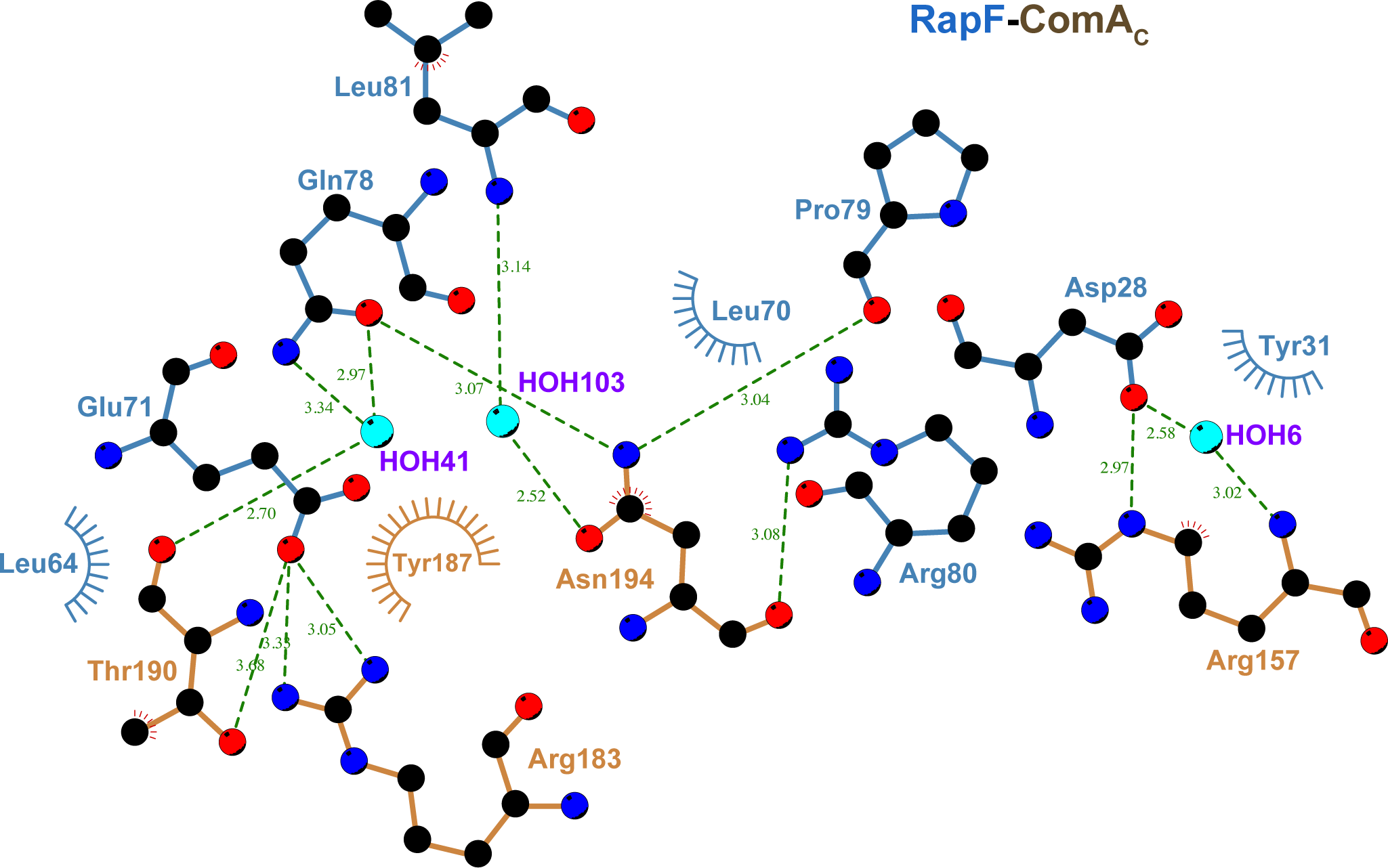

Supplement: Figure S2 — Schematic representation of the ComA residues targeted for mutagenesis and their interactions at the RapF interface. ComA and RapF residues are depicted with brown and blue bonds, respectively. Hydrogen bonds are depicted as dashed green lines. Brown and blue semicircles with radiating lines depict hydrophobic contacts between ComA and RapF residues, respectively. The schematic was produced with LIGPLOT [67]. (TIF) [file pbio.1001226.s002.tif]

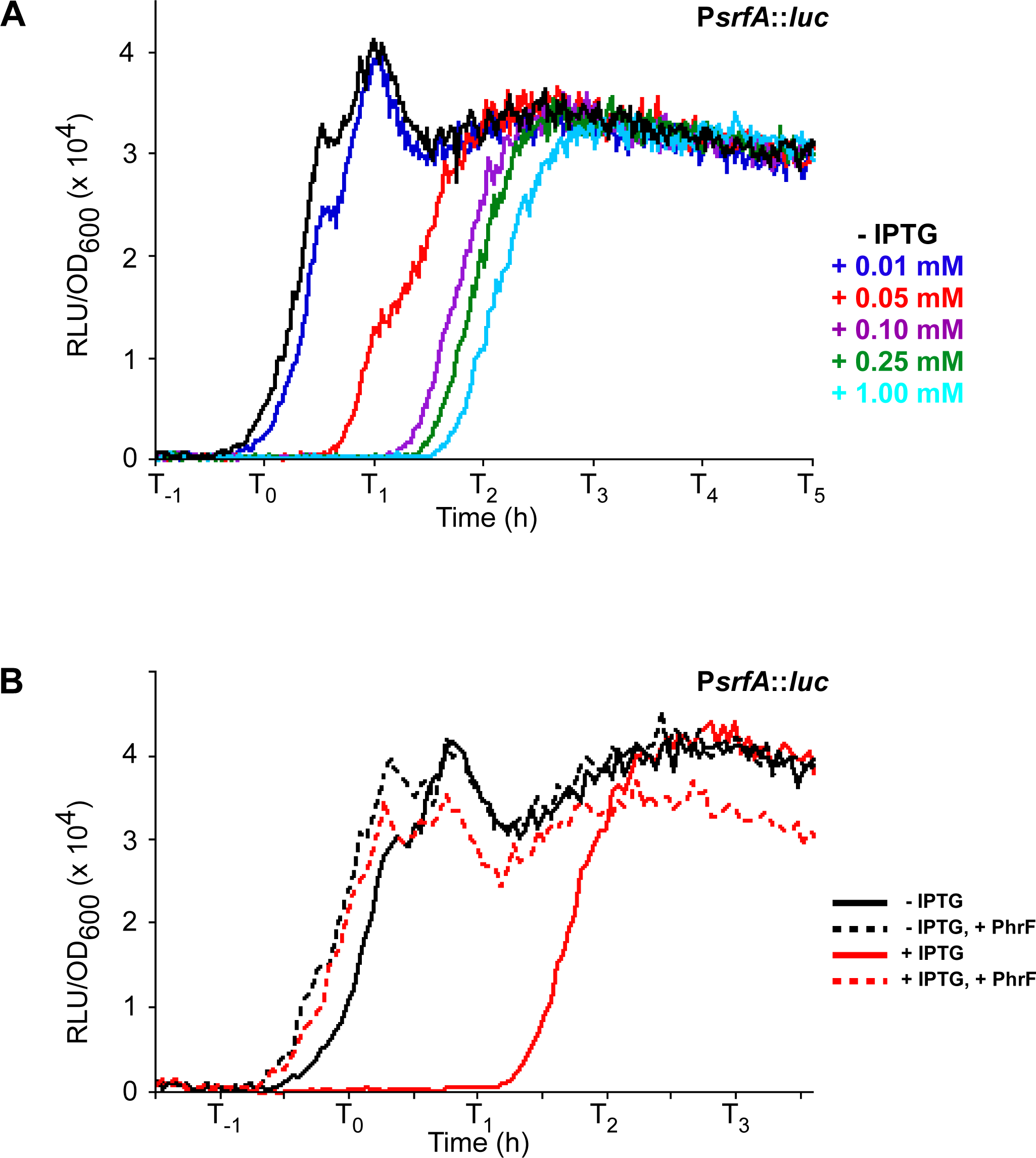

Supplement: Figure S3 — RapF and PhrF regulate the expression of PsrfA-luciferase. (A) PsrfA-luc activity measured in the absence or presence of IPTG at the indicated concentrations. (B) Synthetic PhrF peptide added to the cultures antagonizes the delayed expression of PsrfA-luc caused by the overexpression of RapF. (TIF) [file pbio.1001226.s003.tif]

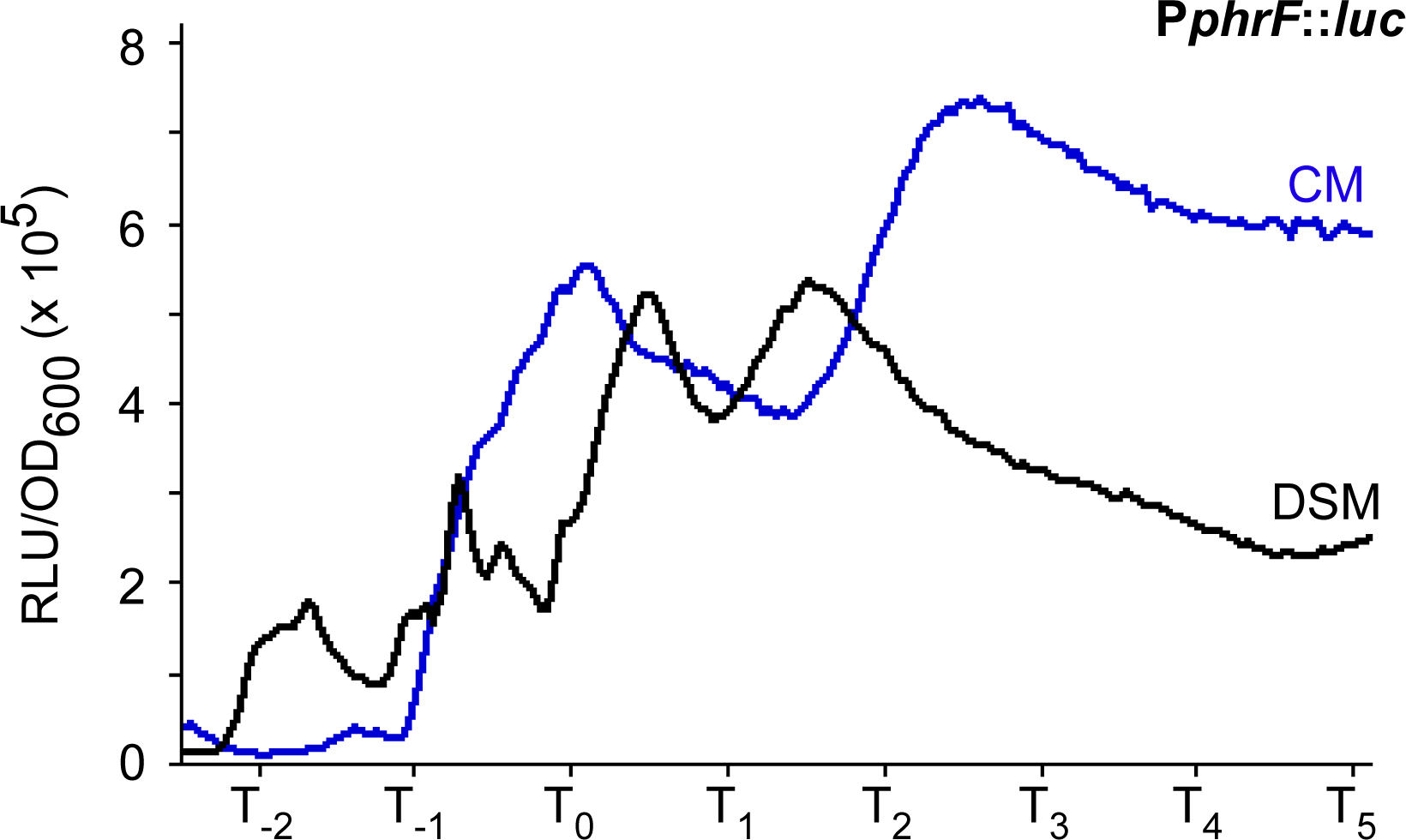

Supplement: Figure S4 — B. subtilis upregulates the expression of phrF during the transition to stationary phase growth (T0). PhrF-luc expression in B. subtilis growing in competence media (CM) or sporulation media (DSM). In addition to being driven by a promoter upstream of the rapF-phrF operon, phrF expression is upregulated during stationary phase by the stationary phase sigma factor, σH, whose binding site lies within rapF [68]. (TIF) [file pbio.1001226.s004.tif]

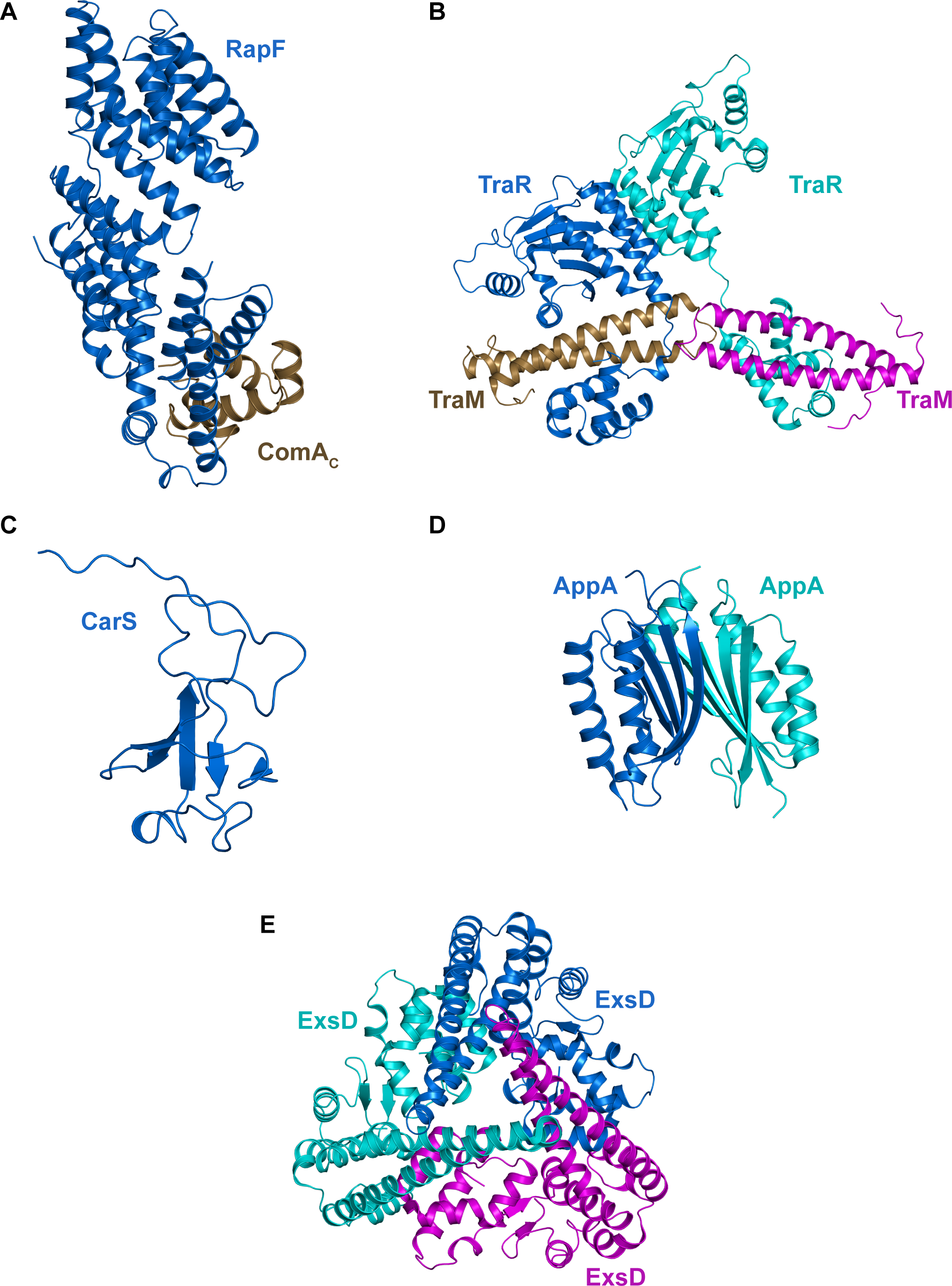

Supplement: Figure S5 — The structures of (A) RapF-ComAC (PDB 3ULQ), (B) TraR-TraM (2Q0O) [35], (C) CarS (2KSS) [31], (D) the AppA BLUF domain (2IYG) [33], and (E) ExsD (3FD9) [34]. To our knowledge the TrlR structure has not yet been determined; however, TrlR is a truncated form of TraR (panel B) [32]. (TIF) [file pbio.1001226.s005.tif]
