# Supplementary material for: Structural Basis of Response Regulator Inhibition by a Bacterial Anti-Activator Protein
Source: PLoS Biol. 2011 Dec 27;9(12):e1001226. doi: 10.1371/journal.pbio.1001226 (PMC3246441; doi:10.1371/journal.pbio.1001226)
Supplement: Table S1 — Data collection and refinement statistics. Rsym = Σh Σi | Ii(h)−<I(h)>|/Σh Σi Ii(h), where Ii(h) is the ith measurement of h and <I(h)> is the mean of all measurements of I(h) for reflection h. Rwork = Σ ||Fo|−|Fc||/Σ |Fo|, calculated with a working set of reflections. Rfree is Rwork calculated with only the test set (5.1%) of reflections. Data for the highest resolution shell are given in parentheses. The structure was determined using a single crystal. (DOC) [file pbio.1001226.s006.doc]

**Table S1.** **Data collection and refinement statistics**

|  | RapF-ComA_C_ |
| --- | --- |
| **Data collection** |  |
| Space group | P2_1_2_1_2 |
| Cell dimensions   |  |
| *a*, *b*, *c* (Å) | 115.30, 81.44, 78.35 |
|     α, β, γ  (°) | 90.00, 90.00, 90.00 |
| Resolution (Å) | 30.00-2.30 (2.34-2.30)^a^ |
| *R*_sym_ or *R*_merge_ | 6.3(61.9) |
| *I* / σ*I* | 27.04(3.28) |
| Completeness (%) | 99.3(100) |
| Redundancy | 7.2(7.4) |
|  |  |
| **Refinement** |  |
| Resolution (Å) | 2.30 |
| No. reflections | 33,197 |
| *R*_work_ / *R*_free_ | 18.88 (25.40)/ 22.41(31.00) |
| No. atoms |  |
| Protein | 3,641 |
| Ligand/ion | 1 |
| Water | 90 |
| *B*-factors |  |
| Protein | 62.23 |
| Ligand/ion | 87.93 |
| Water | 59.71 |
| R.m.s. deviations |  |
| Bond lengths (Å) | 0.008 |
| Bond angles (°) | 1.033 |

^a^Data for the highest resolution shell are given in parentheses. The structure was determined using a single crystal.
