# Supplementary material for: Structural Basis of Response Regulator Inhibition by a Bacterial Anti-Activator Protein
Source: PLoS Biol. 2011 Dec 27;9(12):e1001226. doi: 10.1371/journal.pbio.1001226 (PMC3246441; doi:10.1371/journal.pbio.1001226)
Supplement: Text S1 — Supplemental Materials and Methods. (DOC) [file pbio.1001226.s007.doc]

**SUPPORTING MATERIALS AND METHODS**

**Structural Basis of Response Regulator Inhibition by a Bacterial Anti-Activator Protein**

Melinda D. Baker and Matthew B. Neiditch

**Bacterial Strains and Growth Conditions**

*Escherichia coli* strain DH5α was used for cloning and propagation. *E. coli* strains BL21, BL21(DE3), and BL21(DE3)pLysS were used for protein overexpression. All strains were grown in LB media supplemented with 0.2% glucose when needed and the appropriate antibiotic at the following concentrations: ampicillin, 100 μg/ml; kanamycin, 30 μg/ml or 15 μg/ml; chloramphenicol, 35 μg/ml. All plasmids were introduced into chemically competent *E. coli* strains.

All *Bacillus subtilis* strains were constructed from parental strain IS75 (*hisB2* *leu-8* *metB5*) [1] and grown in competence media (CM) or sporulation media (DSM) [2,3]. Strains harboring a gene cloned downstream of the P*spank(hy)* promoter were grown in the presence of isopropyl-b-D-thiogalactopyranside (IPTG). When needed, the appropriate antibiotics were used at the following concentrations: chloramphenicol, 5 μg/ml; spectinomycin, 100 μg/ml; and erythromycin, 5 μg/ml.

**Plasmid Construction**

An overexpression construct was made to express RapF as an N-terminal glutathione-S-transferase fusion protein (pMDB01) for purification in *E. coli*. Briefly, *rapF* was amplified by PCR from genomic DNA using the following primers: BamHI-RapF-F (5'-CCGGATCCGTGACAGGTGTCATATCTTC-3') and RapF-NotI-R (5'-TGTGCGGCCGCTTAGACTTCAATTTCATACAAACTCAC-3'). The PCR product was digested with BamHI and NotI and ligated to similarly treated pGEX-4T1 (GE Healthcare). pMDB01 was further used as the template for the generation of site-directed alanine mutants as described below. All plasmid constructions were confirmed by DNA sequencing.

An overexpression construct was made to express ComA as an N-terminal hexa-histidine fusion protein, His-ComA, (pMDB07) for purification in *E. coli*. Plasmid pMDB07 was derived from pET28b (Novagen) and PCR amplification of *comA* from genomic DNA using the following primers: pET28-ComA-F-NdeI (5'- CGCGCGGCAGCCATATGAAAAAGATACTAGTGATTGA -3') and pET28-ComA-R-NdeI (5'- GTCATGCTAGCCATATGTTAAAGTACACCGTCTGATT -3'). The PCR product was inserted into NdeI-linearized pET28b using the In-fusion cloning kit according to manufacturer’s guidelines (Clontech). pMDB07 was used as the template for the generation of site-directed alanine mutants as described below.

The ComA C-terminal DNA binding domain (ComA_C_) (residues 146–214), was overexpressed in *E. coli* using pMDB08. pMDB08 was derived from pET28b (Novagen) and PCR amplification of ComA_C_ (nucleotides 436 to 642) from pMDB07 using primers ComA_C_'-NdeI (5'-AAAGCCGCATATGTCCTCTCAAAAAGAACAAG-3') [4] and ComA-R1 (5'-CCGCGAATTCTTAAAGTACACCGTCTGATTTCGC-3'. The PCR product was digested with NdeI and EcoRI and ligated to similarly treated pET28b.

For overexpression of RapF in *B. subtilis*, the entire RapF open reading frame was cloned into pDR111 downstream of the IPTG-inducible P*spank(hy)* promoter [5] using the following primers: pDR111-rbsRapF-F (5'-ATTAAGCTTAGTCGACTTTTTGCGCTTCTCACAAG-3') and RapF-SphI-pDR111-R (5'-CACCGAATTAGCTTGCATGCTTAGACTTCAATTTCATAC-3') and inserted into SalI/SphI digested pDR111 using the In-fusion cloning kit (Clontech). pMDB26 was used as the template for generation of site-directed alanine mutants (described below).

**Generation of Alanine-Substituted RapF and ComA Mutants**

RapF and ComA mutant expression vectors were generated by site-directed mutagenesis of pMDB01, pMDB26, and pMDB07 using the ChangeIT Mutagenesis Kit (USB). The expression vectors contained only the desired mutations as determined by DNA sequencing.

**Protein Purification of RapF and ComA for In Vitro Studies**

Wild-type and alanine-substituted RapF mutants were overexpressed in *E. coli* strain BL21 as described for the proteins used for crystallization experiments. Protein expression was induced, and cultures were harvested and lysed in the presence of Buffer I as described for crystallization experiments. The RapF proteins were then purified by

affinity chromatography using Glutathione-Uniflow Resin (Clontech), and the GST tag was cleaved with thrombin. Fresh Glutathione-Uniflow Resin was used to separate RapF from the GST tag and any contaminating uncleaved GST-RapF. The proteins were collected and concentrated by ultracentrifugation (MWCO: 30 kD; Amicon).

Wild-type ComA, wild-type His-ComA, and the His-ComA alanine-substituted mutants were overexpressed in *E. coli* strain BL21(DE3)pLysS grown in LB media supplemented with 15 μg/ml kanamycin, 35 μg/ml chloramphenicol, and 0.2% (w/v) L-glucose at 37 °C to an OD_600_ of 0.800. Protein expression was induced with 1.0 mM IPTG at 37 °C for 5 h. Cells were harvested by centrifugation at 4,000 RPM, lysed in Buffer IV (20 mM Tris pH 8.0, 300 mM NaCl, 5 mM MgCl_2_, 10 mM β-mercaptoethanol, and 5% glycerol) supplemented with 25 mM imidazole, 20 μg/ml DNaseI, and 2 mM phenylmethylsulfonyl fluoride, and insoluble material pelleted at 22,000 RPM for 90 minutes. Cleared lysates were applied to His60 Ni Superflow Resin (Clontech) equilibrated with Buffer IV containing 25 mM imidazole and then washed with Buffer IV containing 50 mM imidazole. ComA was eluted with a 50-500 mM imidazole gradient. ComA containing fractions were pooled, diluted 6-fold with Buffer V (20 mM Tris pH 8.0, 5 mM MgCl_2_, 5 mM DTT, and 5% glycerol), passed through a 0.22 μm filter, and applied to an anion exchange column (MonoQ HR5/5; Pharmacia) equilibrated with Buffer V containing 50 mM NaCl. ComA was eluted in a 50-850 mM NaCl linear gradient of the same buffer. ComA containing fractions were pooled and concentrated by ultracentrifugation (MWCO: 10 kD; Amicon).

Wild-type ComA used for native PAGE analysis was treated with 3.5 μg/ml thrombin overnight at 4 °C to remove the N-terminal His affinity tag. ComA was then passed over fresh His60 Ni resin to remove the His tag and residual uncleaved His-ComA. ComA was then further purified by anion exchange chromatography as described above. The wild-type His-ComA used for analytical gel filtration studies was further purified by gel filtration using a Superdex 200 (GE Healthcare) 16/70 column equilibrated with Buffer V containing 300 mM NaCl.

**Construction of *B. subtilis* Luciferase Promoter Fusion Strains**

A P*phrF* luciferase gene fusion was integrated into the *thrC* locus. The P*phrF* fragment was cloned upstream of the *Photinus pyralis* luciferase gene and inserted into the multiple cloning site of pDG1664 [6], which carries an erythromycin resistance cassette and fragment of *hom-thrC* and *thrC-thrB* from pSU11, in two steps to yield pMDB36 (P*phrF*-*luc*). First, the *phrF* promoter fragment [7] was amplified using the following primer pair: pDG1664-BamHI-phrF-F (5'- CTGCCTTCGGATCCGAAAGCAAGCTGATAT-3') and PphrF-NcoI-luc-R (5'- GGTCCATGGACTTCAATTTCATACAAACTC-3'). The luciferase ORF was PCR amplified from pGL3 (Promega) using NcoI-Luc-F (5'-TGAGAATGGAGGAGGACACCATGGAAGACGCCAA-3') and pDG1664-Luc-EcoRI-R (5'-TAGGGTTATCGAATTCTTACACGGCGATCTTTCCGC-3'). PCR amplified P*phrF* and the *luc* genes were digested with NcoI and ligated. The final insert for plasmid pMDB36 was generated by PCR amplification of the ligation mixture using the forward and reverse primers pDG1664-BamHI-pphrF-F and pDG1664-Luc-EcoRI-R. This insert was then cloned into BamHI/EcoRI-digested pDG1664 using the In-fusion cloning kit (Clontech). P*phrF-luc* was integrated into the *thrC* locus of IS75 by homologous recombination to give MN1105 *thrC*::P*phrF* (er).

A P*srfA-luc* reporter strain (BD4798 – a generous gift from N. Mirouze) contains a 1 kb 5' fragment of the *srfA* operon including the initiation codon and the promoter and the *luc* open reading frame from pGL3. P*srfA-luc* was introduced into *B. subtilis* by a single crossover event at the native *srfA* locus, reconstructing the endogenous regulatory region in front of the fusion and a complete copy of the *srfA* operon downstream of the fusion. pMDB26-derived plasmids were then transformed into BD4798 P*srfA::luc (cm)*, which by double-crossover recombination at the *amyE* locus yields MN1075-derived strains P*srfA::luc* (cm), *amyE*::P*_spank(hy)_rapF* (spc) expressing wild-type or mutant *rapF* under control of the IPTG-inducible hyperspank promoter,

**Luciferase Assays**

Luciferase assays were performed as previously described with the following modifications [8]. The *PsrfA-luc* reporter strain (MN1075) and P*phrF*-*luc* reporter strain (MN1105) were grown in LB medium to an OD_600_ of 2.0, centrifuged, and resuspended in fresh CM or DSM [2] to an OD_600_ of 2.0. The cultures were then diluted 20-fold in fresh media supplemented with IPTG and synthetic PhrF (LifeTein) where indicated. 200 μl were dispensed per well in duplicate in a 96-well black plate (Corning). Luciferin was added to each well at a final concentration of 0.47 μM. The cultures were then incubated at 37 °C under agitation in a PerkinElmer Envision 2104 Multilabel Reader. The plate lids were heated to 38 °C to avoid condensation. Relative Luminescence Unit (RLU) and OD_600_ were measured at 1.78 min intervals.

**SUPPORTING REFERENCES**

1. Hahn J, Dubnau D (1991) Growth stage signal transduction and the requirements for srfA induction in development of competence. J Bacteriol 173: 7275-7282.

2. Albano M, Hahn J, Dubnau D (1987) Expression of competence genes in Bacillus subtilis. J Bacteriol 169: 3110-3117.

3. Schaeffer P, Millet J, Aubert JP (1965) Catabolic repression of bacterial sporulation. Proc Natl Acad Sci U S A 54: 704-711.

4. Bongiorni C, Ishikawa S, Stephenson S, Ogasawara N, Perego M (2005) Synergistic regulation of competence development in Bacillus subtilis by two Rap-Phr systems. J Bacteriol 187: 4353-4361.

5. Ben-Yehuda S, Rudner DZ, Losick R (2003) RacA, a bacterial protein that anchors chromosomes to the cell poles. Science 299: 532-536.

6. Guerout-Fleury AM, Frandsen N, Stragier P (1996) Plasmids for ectopic integration in Bacillus subtilis. Gene 180: 57-61.

7. McQuade RS, Comella N, Grossman AD (2001) Control of a family of phosphatase regulatory genes (phr) by the alternate sigma factor sigma-H of Bacillus subtilis. J Bacteriol 183: 4905-4909.

8. Parashar V, Mirouze N, Dubnau DA, Neiditch MB (2011) Structural basis of response regulator dephosphorylation by rap phosphatases. PLoS Biol 9: e1000589.
